# Systematic analysis of the *Capsicum* ERF transcription factor family: identification of regulatory factors involved in the regulation of species-specific metabolites

**DOI:** 10.1186/s12864-020-06983-3

**Published:** 2020-08-24

**Authors:** Jiali Song, Changming Chen, Shuanglin Zhang, Juntao Wang, Zhubing Huang, Muxi Chen, Bihao Cao, Zhangsheng Zhu, Jianjun Lei

**Affiliations:** 1 Key Laboratory of Biology and Genetic Improvement of Horticultural Crops (South China), Ministry of Agriculture and Rural Affairs, College of Horticulture, South China Agricultural University, Guangzhou, Guangdong 510642 People’s Republic of China; 2 Lingnan Guangdong Laboratory of Modern Agriculture, Guangzhou, 510642 China; 3 Guangdong Helinong Seeds, CO.LTD, Shantou, 515800 Guangdong China; 4 Peking University-Southern University of Science and Technology Joint Institute of Plant and Food Sciences, Department of Biology, Southern University of Science and Technology, Shenzhen, 518055 China; 5 Henry Fok College of Biology and Agriculture, Shaoguan University, Shaoguan, 512005 China

**Keywords:** Pepper, ERF, Carotenoids, Capsaicinoids, Temperature

## Abstract

**Background:**

ERF transcription factors (TFs) belong to the Apetala2/Ethylene responsive Factor (AP2/ERF) TF family and play a vital role in plant growth and development processes. Capsorubin and capsaicinoids have relatively high economic and nutritional value, and they are specifically found in *Capsicum*. However, there is little understanding of how ERFs participate in the regulatory networks of capsorubin and capsaicinoids biosynthesis.

**Results:**

In this study, a total of 142 ERFs were identified in the *Capsicum annuum* genome. Subsequent phylogenetic analysis allowed us to divide ERFs into DREB (dehydration responsive element binding proteins) and ERF subfamilies, and further classify them into 11 groups with several subgroups. Expression analysis of biosynthetic pathway genes and *CaERFs* facilitated the identification of candidate genes related to the regulation of capsorubin and capsaicinoids biosynthesis; the candidates were focused in cluster C9 and cluster C10, as well as cluster L3 and cluster L4, respectively. The expression patterns of *CaERF82*, *CaERF97*, *CaERF66*, *CaERF107* and *CaERF101*, which were found in cluster C9 and cluster C10, were consistent with those of accumulating of carotenoids (β-carotene, zeaxanthin and capsorubin) in the pericarp. In cluster L3 and cluster L4, the expression patterns of *CaERF102*, *CaERF53*, *CaERF111* and *CaERF92* were similar to those of the accumulating capsaicinoids. Furthermore, *CaERF92*, *CaERF102* and *CaERF111* were found to be potentially involved in temperature-mediated capsaicinoids biosynthesis.

**Conclusion:**

This study will provide an extremely useful foundation for the study of candidate ERFs in the regulation of carotenoids and capsaicinoids biosynthesis in peppers.

## Background

Peppers (*Capsicum* spp.) including sweet and chili varieties, are among the most economically important crops in the world. Carotenoids pigments and pungency are important, typical characteristics of peppers, and the accumulation of carotenoids pigments affects the intensity of coloration (red, yellow and orange color) in ripe *Capsicum* fruit. Carotenoids pigments are synthesized in plastids and are generated from the prenyl lipid biosynthesis pathway (Fig. S1A). In the final step, geranylgeranyl pyrophosphate (GGPP), the prenyl lipid precursor, is transformed into capsorubin or capsanthin through a series of enzymatic actions mediated by phytoene synthase (PSY), phytoene desaturase (PDS), and lycopene β-cyclase (LCYB) [1, 2]. The colour and multi-nutritional content of pepper are principally attractive features that depend on carotenoids. Capsanthin and β-carotene uptake from peppers is the most common in humans among carotenoids [2]. Carotenoids not only possess potent antioxidant activity but also provide potential benefits for immunity and diseases, such as certain cancers, cardiovascular diseases and eye disease [3–6]. Moreover, one of the most important characteristics of pepper fruit is pungency, which is a result of the accumulation of capsaicinoids, which are alkaloids. Capsaicinoids biosynthesis is unique to *Capsicum* spp., and it is characterized by tissue specificity. Capsaicinoids biosynthesis occurs in the epidermis of the placenta, and capsaicinoids are stored in vesicles on the surface of this tissue and the pericarp [7]. Previous studies have reported that more than 23 types of capsaicinoids were found in peppers [8, 9]. Both capsaicin and dihydrocapsaicin were the most abundant capsaicinoids, representing 91% of the total capsacinoids content [10]. The biosynthetic pathway of capsaicinoids consists of two distinct pathways, the phenylalanine and chain fatty acid biosynthesis pathways, which are involved in a series of genes encoding enzymes involved in synthesis, such as Phe ammonia-lyase (Pal), caffeic acid O-methyltransferase (Comt) and a putative acyltransferase (AT3) (Fig. S1B) [11–13].

TFs are closely associated with developmental processes and responses to environmental stimuli. The ERF family belongs to the largest branch of the AP2/ERF superfamily. Its members are characterized by having a highly conserved AP2 domain that is 60–70 amino acids, which is located in the DNA-binding region [14]. Based on the binding of *cis*-acting elements to promoters, the ERF family is further classified into two subfamilies, ERF and DREB families [15, 16]. The activity of ERF TFs depends on the AP2 domain binding *cis*-acting elements in the promoter regions of their target genes. For example, in the ERF subfamily, the genes in the promoter region specifically bind to the additional nucleotide acid sequence AGCCCGCC of the GCC-box, while the members of the DREB subfamily typically bind to a core sequence CCGAC which belongs to a component of dehydration-responsive element-binding [15, 16].

ERF TFs play a critical role in plant development and stress responses, such as cell wall formation [17], fruit ripening [18] and response to cold, salt, drought and resistance and defense against numerous diseases [19–22]. ERF TFs specifically participate in primary and secondary metabolism of the plant as well, which includes the production of steroidal glycoalkaloids [23, 24], anthocyanin [25] and carotenoids [18]. Numerous studies have revealed that TFs are closely associated with capsaicinoids biosynthesis [13, 26, 27]. The members of the ERF family are TFs that are candidates for the control capsaicinoids biosynthesis. *PAL* genes possess a homologue of the GCC-box in their promoters, and *ERF* genes can combine with this cis-acting element [28, 29]. In peppers, capsaicinoids-related biosynthetic genes, including *Acl*, *FatA* and *C4H*, possess the specific sequence CCTTAGA, which was also can be recognized by JERF [30]. The expression levels of *Erf* and *Jerf* were found to be closely related to pungency in nine pepper cultivars with distinct capsaicinoid contents, and they were expressed at high levels at 16–20 days post-anthesis (DPA), which was in consistent with the expression of key capsaicinoids biosynthetic genes [31]. *Erf* and *Jerf* therefore presumably participate in the capsaicinoids biosynthetic pathway. Additionally, ERF TFs have been reported to associate with carotenoids synthesis pathways in some plant species, such as tomato, papaya and *Arabidopsis* [18, 32–34]. However, a clear understanding of how carotenoids and capsaicinoids biosynthesis is regulated at the level of transcription is currently unknown in peppers.

Carotenoids pigments and capsaicinoids not only are typically important characteristics of for *Capsicum* ripe fruit but also are widely applied in medicine, military and chemical industry areas. However, the biological functions of ERF TFs to regulate carotenoids and capsaicinoids biosynthesis are unknown. To obtain an understanding of the role of the ERF family in transcriptionally modulating carotenoids and capsaicinoids biosynthesis, the all members of the ERF family were characterized by utilizing the newly sequenced *Capsicum annuum* genome. Characteristic analysis was carried out to identify the involvement of specific ERF family members in carotenoids and capsaicinoids biosynthesis. Overall, this study contributed to the understanding of the function of ERF family members in the carotenoids and capsaicinoids biosynthetic pathways in peppers. Capsaicinoids biosynthesis is affected by environmental factors. Therefore, the function of candidate ERF TFs associated with capsaicinoids biosynthesis was also analysed in response to different temperatures.

## Results

### Identification and multiple sequence alignment of CaERF proteins in pepper

A total of 142 *ERF* genes were obtained from the *Capsicum annuum* genome after excluding redundant sequences, the candidates containing an AP2 plus a B3 domain, and candidates containing more than two AP2 domains (Table S3). The 142 candidate genes were renamed consecutively according to the chromosomal positions (Table S3; Fig. S2). In addition, all identified ERF members encoded 44–672 residues. The molecular weight (Mw) of each CaERF protein ranged from 7.19 kDa to 74.91 kDa, and the theoretical pI varied from 4.24 to 11.10. Most of these proteins were unstable, and only fifteen CaERF proteins were stable (instability index< 40) (Table S3).

**Fig. 1:**
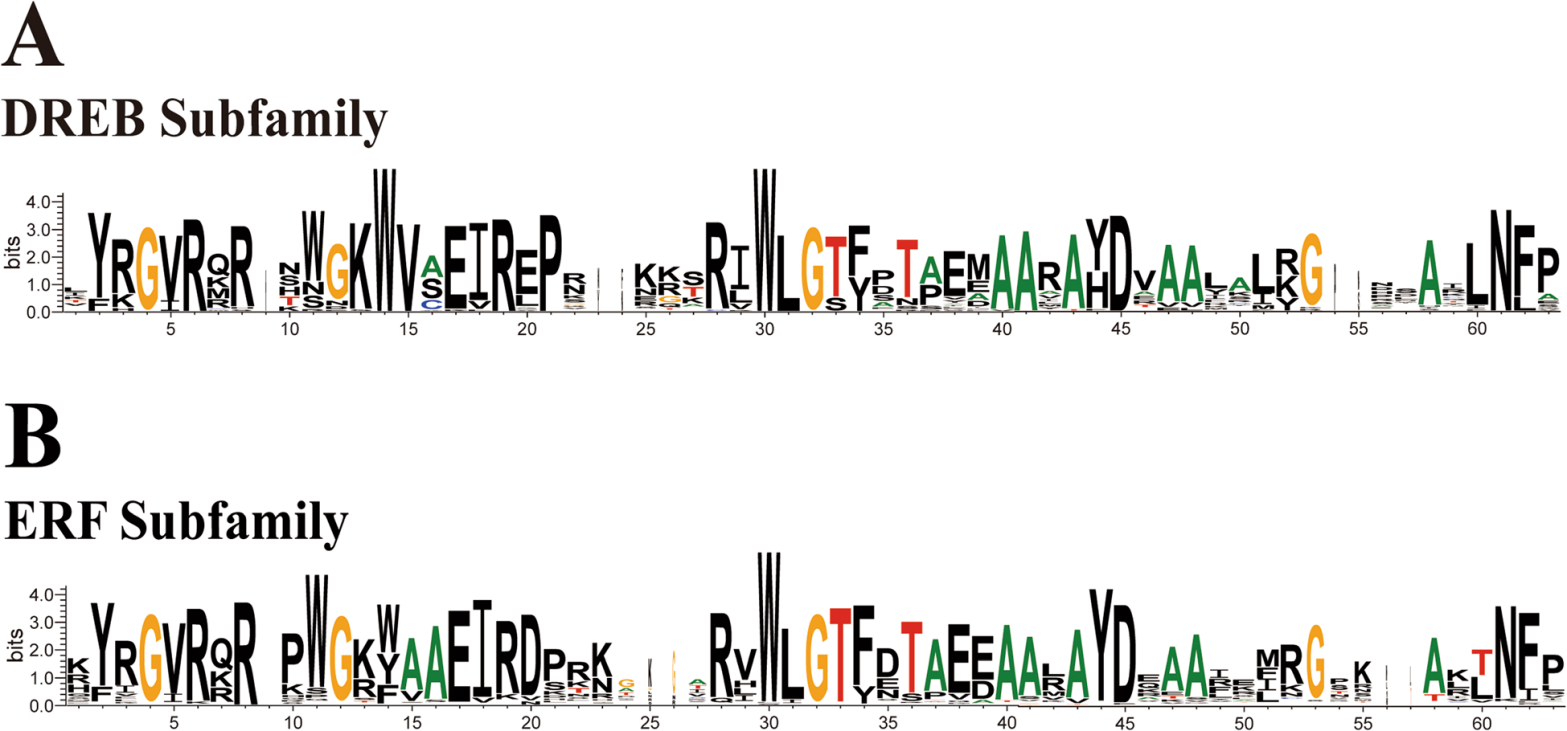
Sequence representation of LOGO derived from multiple sequence alignment of DREB (**a**) and ERF (**b**) subfamily. The height of the amino acid indicates the frequency observed in multiple alignment

**Fig. 2:**
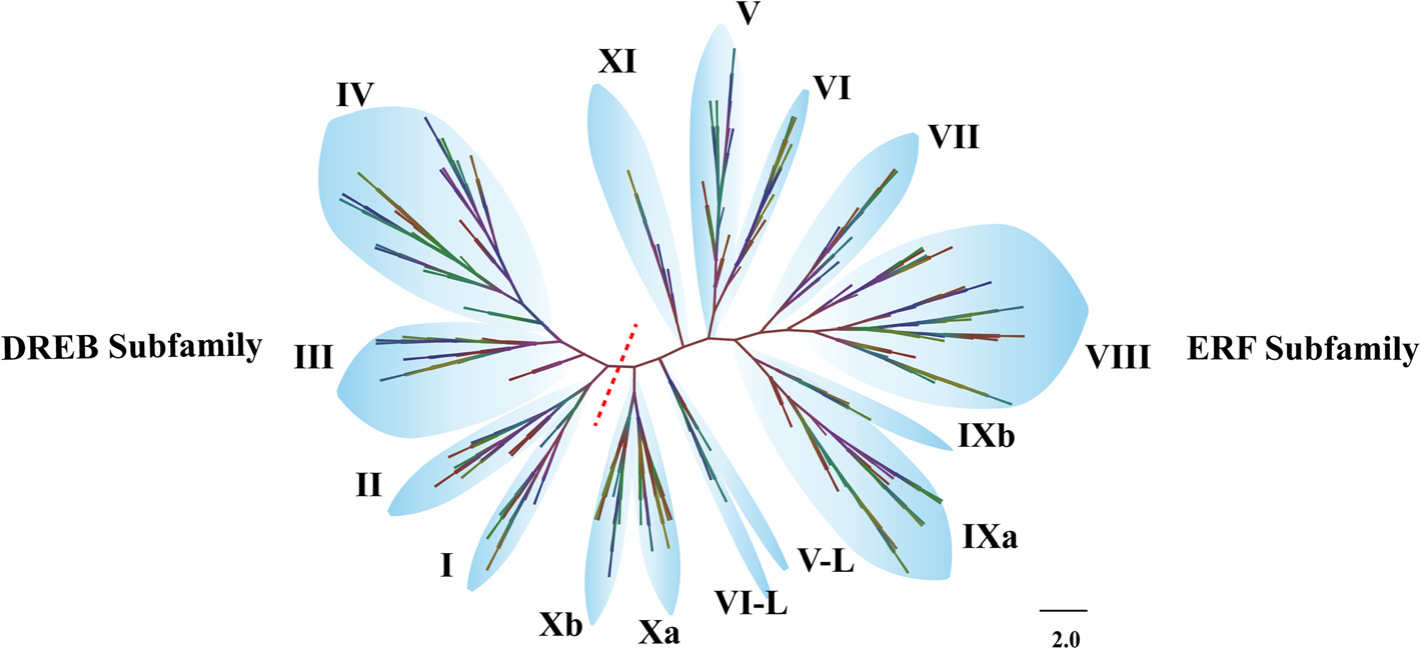
Neighbour-joining phylogeny of the pepper ERF family in relation to *Arabidopsis*. The groups were named and classified according to *Arabidopsis* [15]. The DREB and ERF subfamilies are divided by the dashed red line. Both groups X and XI possessed very low conservation in the 15th and 20th amino acids, and they were near the dashed red line. Groups X and XI were tentatively defined as the ‘ERF-like subfamily’

Before phylogeny analysis was performed, multiple alignment analyses were performed using the amino acid sequences of the AP2 domains. The classification of all identified CaERFs is shown in Fig. 2, as described later. The alignment analyses indicated that the DREB subfamily possesses a specific WLG motif that is a completely conserved residue (Fig. 1a; Fig. S3), while more than 95% of members in the ERF subfamily had a WLGT motif for the ERF subfamily except for groups X and XI (Fig. 1b; Fig. S3). The DREB subfamily was completely conserved in V15 and E20, and more than 95% of the members of groups V to IX in the ERF subfamily contained A14 and D19 (Fig. 1ab; Fig. S3). The shaded residues shown for 37 DREB subfamily members indicate complete conservation in the AP2 domain (Fig. 1a; Fig. S3). However, the alignment revealed that the N-terminal regions of the AP2 domains in the ERF subfamily possessed a high homology, while those of the C-terminal regions showed very low conservation (Fig. 1b; Fig. S3). Moreover, groups X and XI possessed very low conservation in the 15th and 20th amino acids, and there was difficulty in in classifying these residues. Nevertheless, taking into account the topology of the tree in Fig. 2, groups X and XI were preliminarily classified as the ‘ERF-like subfamily’.

### Phylogenetic analysis of the ERF family in four plant species

To clarify the phylogenetic relationships, an unrooted phylogenetic tree was constructed for all of the identified CaERF sequences based on their alignment with those in *Arabidopsis* by a neighbour-joining phylogenetic analysis. As shown in Fig. 2, based on the classification of *AtERF* in Nakano’s and Sakuma’s studies [15, 16], putative CaERF proteins were divided into two large subfamilies that corresponded to the DREB and ERF subfamilies (Fig. 2; Fig. S4). According to the cited studies [15] and taking into account the topology of the tree, the two subfamilies were further defined as 11 groups named group I to XI (Table 1; Fig. S4).

**Table. 1:** Summary of each group of ERF families in four plant species

Subfamily	Group	Pepper	***Arabidopsis***	Rice	Tomato
**DREB**		**37**	**57**	**54**	**43**
	I	5	10	9	7
	II	8	15	9	7
	III	5	23	13	7
	IV	19	9	23	22
**ERF**		**107**	**65**	**84**	**94**
	V	11	5	13	12
	VI	5	8	2	4
	VII	7	5	9	5
	VIII	31	15	19	35
	IXa	13	17	20	15
	IXb	5	0	1	0
	Xa	11	8	8	13
	Xb	12	0	0	2
	V-L	0	3	0	0
	VI-L	2	4	1	4
	XI	8	0	11	4
	**Total**	**142**	**122**	**138**	**137**

Notably, some differences existed in groups IX and X, which were then subdivided into IXa, IXb, Xa and Xb, because the members of groups IXb and Xb were only found in peppers. Additionally, the members of group XI were present only in pepper as well, whereas group V-Like (V-L) were absent in pepper (Table 1). These results indicated that the members of IXb, Xb and XI might be pepper-specific groups. To determine whether these three groups were specific to peppers, all *CaERF* genes were used to construct a neighbour-joining phylogenetic tree with those from tomato (137), rice (138) and *Arabidopsis* (Fig. S5). The topology of the phylogeny was mostly similar to that tree obtained when using only protein sequences from pepper and *Arabidopsis* (Fig. S4). The number of ERF proteins in each group is listed in Table 1. Groups IXb and Xb contained a significantly higher number of ERF TFs from peppers. In contrast, the members of ERF members in group XI included rice and tomato, and no significant differences were observed in other investigated species (Table 1). Therefore, groups IXb and Xb were designated as putative ‘pepper-specific groups’ (Fig. 2).

To evaluate the biological functions of the CaERF protein of the groups, the functional characteristics of ERF from *Arabidopsis*, tomato and pepper were investigated in the literature. As shown in Table S4, the members of the same group possessed similar biological functions, and group VIII members were found to be likely involved in alkaloid biosynthesis. Because of the importance of capsaicinoids and capsorubin in pepper, the possibility of the *Capsicum annuum* genome (version 2.0) containing putative ERF homologs involved in secondary metabolites was investigated. A previous study demonstrated that *Erf* and *Jerf* in peppers were involved in the regulation of the pungency phenotype [31]. Erf and Jerf were mapped to CaERF53 and CaERF101 in the *Capsicum annuum* genome (version 2.0), respectively. (Table S5). Moreover, CaERF101 was identified as the putative orthologue of both CaPF1 and JERF1, and it was shown to be associated not only with the regulation of polyamine biosynthesis but also with ABA biosynthesis (Table S5). It was likely that the members of group VII which contained CaERF53 and CaERF101, were related to secondary metabolite biosynthesis.

### Conserved motif analysis of CaERF

**Fig. 3:**
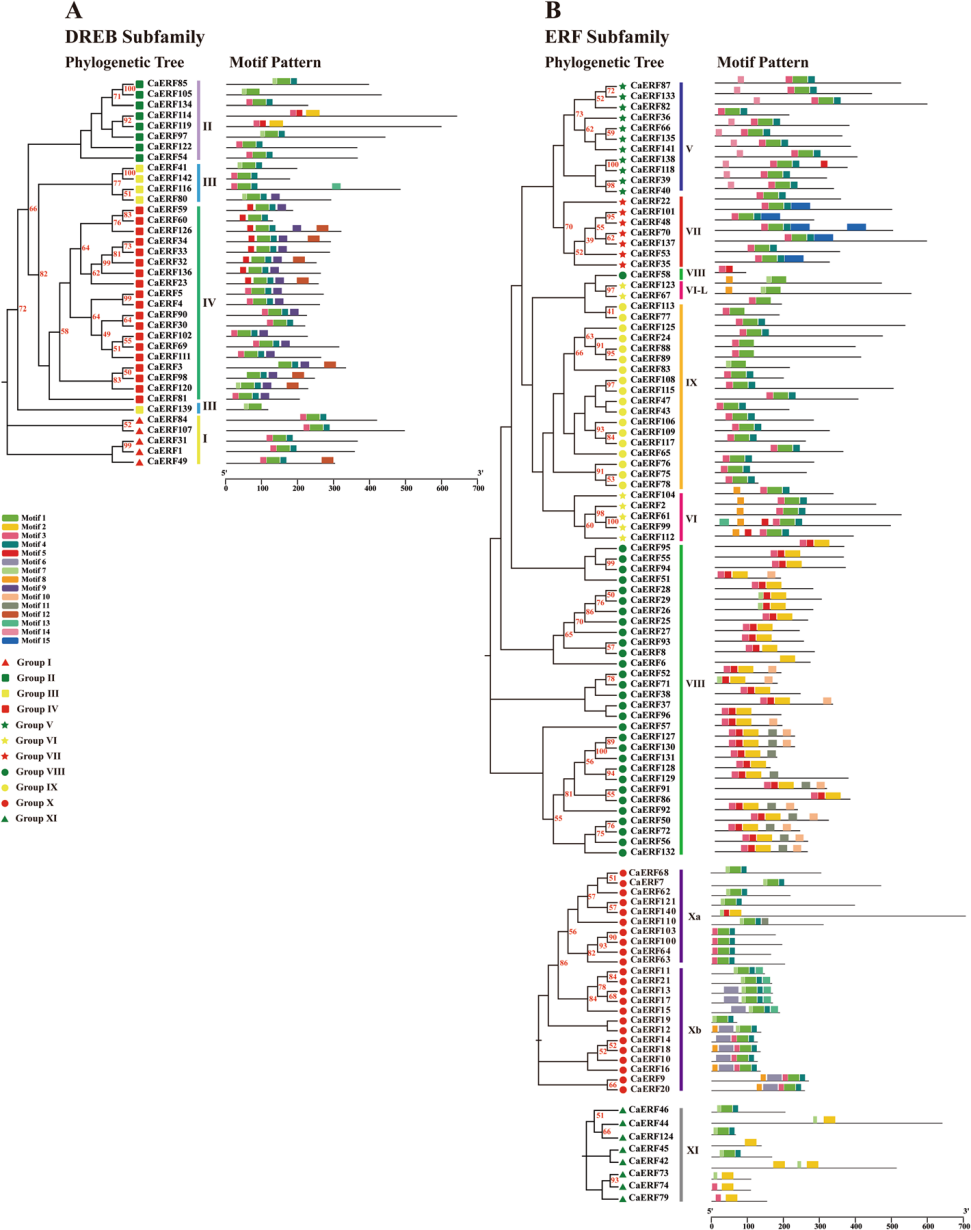
Phylogenetic relationships and conserved motif distributions of the DREB (**a**) and ERF (**b**) subfamilies of proteins. **a** TFs from group I to group IV were used to construct a phylogenetic tree. **b** TFs from group V to group IX, group X and group XI were separately used to construct a phylogenetic tree. A phylogenetic tree was constructed using the neighbour-joining method. Different coloured boxes indicate different motifs. Box length was equivalent to motif length. Different color shapes indicated different groups in the phylogeny of CaERFs associated with *Arabidopsis*

Conserved amino acid motifs represent functional areas maintained during the evolutionary process. The conserved motifs within the 142 CaERF sequences were analysed and compared using MEME. A total of 15 significantly conserved motifs (E-value < 10^− 32^) possessing 11–41 residues were identified and named motif 1 to motif 15 (Table S6). Five conserved amino acid motifs, motif 1 to motif 5, were found to be located in the AP2 domain region, which were present in the majority of CaERF proteins and designated as “general motifs” (Fig. 3); however, both motif 2 and motif 5 were mainly shared within group VIII in the ERF subfamily (Fig. 3b). The remaining motifs (motif 6 to motif 15) were distributed outside of the AP2 domain and were classified as “specific motifs”. Motif 9 and motif 12 were primarily restricted to group IV in the DREB subfamily (Fig. 3a). Motif 10 and motif 11 were specifically contained in group VIII. Motifs 6 and 13 were found in group X, and motif 14 was in group V (Fig. 3b). Further, motif 15 was specifically present in group VII. Nevertheless, the same group of trees harboured similar motif patterns (Fig. 3).

### Expression patterns of *CaERFs* in different developmental stages of pericarp and placenta

**Fig. 4:**
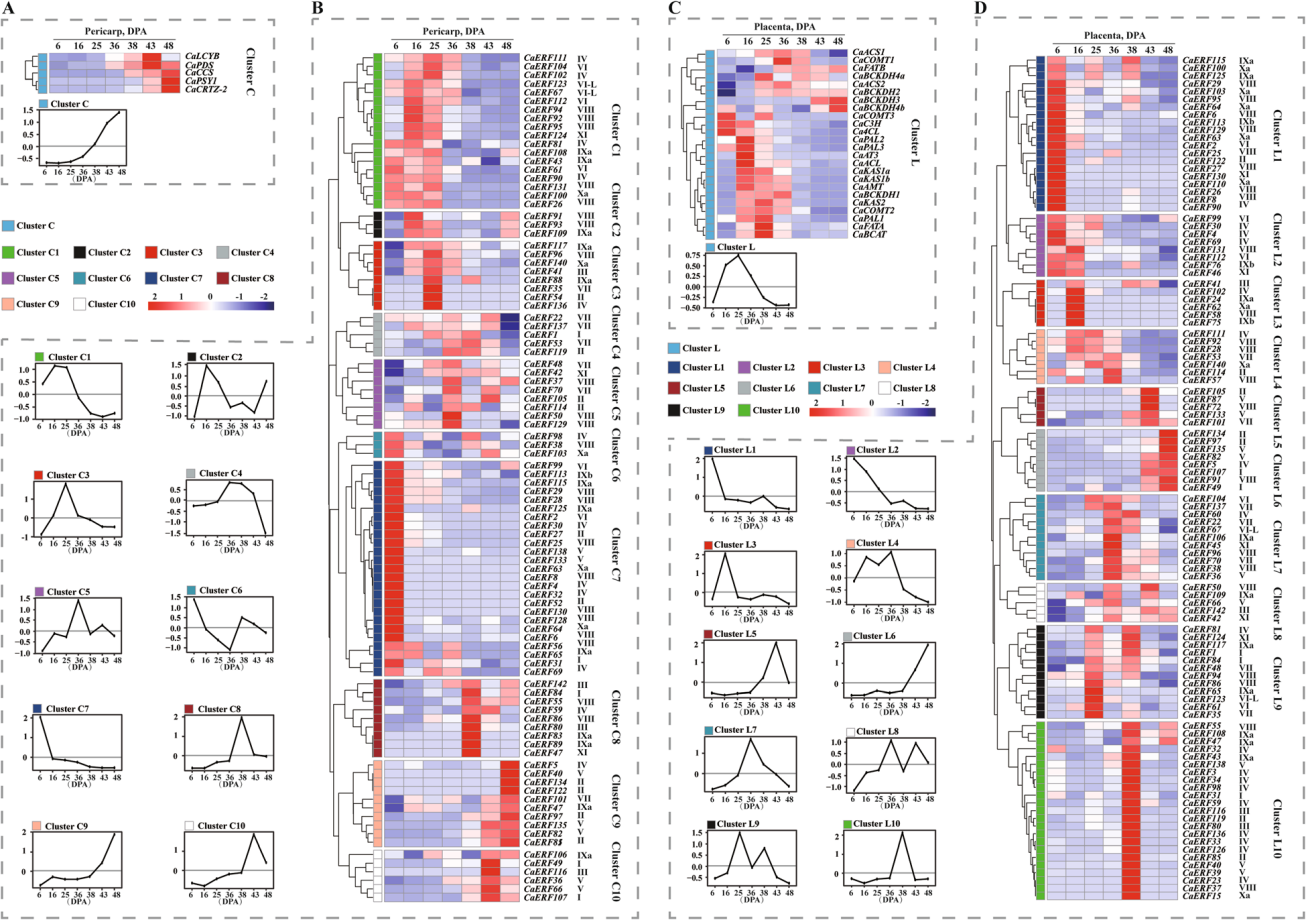
Transcript abundance of genes involved in capsanthin/capsaicinoids and biosynthesis and *CaERF* genes in the pericarp and placenta at different developmental stages. **a** and **b** indicate the expression patterns of genes involved in capsanthin biosynthesis and *CaERF* genes, respectively, in the pericarp at different developmental stages. **c** and **d** show the expression patterns of genes involved in capsaicinoidsoid biosynthesis and *CaERF* genes in placenta at different developmental stages, respectively. The heat map was constructed by log2 values of fragments per kilobase of exon per million fragments mapped (FPKM). The name of each gene with the name of the phylogenetic group is shown at the right of the heat map. Line charts were made using the mean value for the whole cluster. The letter “C” in cluster C represents the pericarp. The letter “L” in cluster L represents the placenta

Expression patterns imply a biological function for genes. Capsorubin and capsaicinoids are specifically biosynthesized in pepper fruit, and their accumulation pattern is regulated through developmental stages. To gain further insight into the hypothetical roles of CaERFs during the capsorubin and capsaicinoids biosynthesis processes, the expression patterns of *CaERFs* and the genes involved in synthesis in the pericarp and placenta (including 6, 16, 25, 36, 38, 43, and 48 DPA stages) were investigated (Fig. 4). RNA-Seq raw data were retrieved from a public database [13] and all of the reads were remapped to the *Capsicum annuum* genome (version 2.0). The expression of the relevant capsorubin synthesis gene gradually increased at 36 DPA, and capsorubin itself primarily accumulated at this stage (Fig. 4a, cluster C). A total of 48 *CaERF* (33%) transcripts were expressed at a level that could not be detected. Based on similar expression patterns, the expression patterns of *CaERF* in the pericarp were hierarchically clustered, and divided into 10 clusters (Fig. 4b). The expression of members of cluster C9 and cluster C10 was in agreement with the transcriptional trend of relevant-capsorubin synthetic genes. Although the expression of members of cluster C10 gradually decreased after 43 DPA, the genes involved in the synthesis of capsorubin (i.e., *CaPDS* and *CaLCYB*) were also gradually expressed after this stage. This result indicated that these ERF TFs may regulate different genes involved in capsorubin biosynthesis. Thus, the members of cluster C9 and cluster C10 were candidates for the regulation of capsorubin biosynthesis.

The expression of genes involved in capsaicinoids synthesis tended to rapidly increase from 6 DPA to 25 DPA, and then they gradually decreased, which was consistent with abundant production of capsaicinoids at stages from 13 DPA to 25 DPA (Fig. 4c). A total of 38 *CaERFs* (26%) were expressed at a level that could not be detected in any of the developmental stages of the placenta. The placenta-expressed genes were hierarchically clustered based on similar expression patterns, yielding 10 clusters (Fig. 4d). Generally, *CaERF* in the same phylogenetic group revealed distinct expression. In the ten clusters, only the expression of members in cluster L3 and cluster L4 exhibited good agreement with the stages of abundant-capsaicinoids accumulation. However, the expression of cluster L3 members (*CaERF85*, *CaERF101*, *CaERF65* and *CaERF73*) was high at 6 DPA, and then it was not detected at other stages, with the exception of *CaERF116* and *CaERF102*. The transcript level of cluster L4 members (*CaERF111*, *CaERF92*, *CaERF28*, *CaERF53*, *CaERF103*, *CaERF114*, *CaERF25* and *CaERF139*) increased from 6 DPA to 36 DPA, but levels slightly decreased at 25 DPA. A previous study demonstrated that CaERF53 and CaERF101 were related to capsaicinoids biosynthesis [31]. However, CaERF101 was included in cluster L5, and the members of this cluster exhibited increased transcript levels at 38 DPA. Therefore, the members of cluster L3 and cluster L4 were represent novel candidates for the regulation of capsaicinoids biosynthesis.

Additionally, the members of two putative ‘pepper-specific groups’ (IXb and Xb) were barely expressed during all of the developmental pericarp and placenta stages, with the exception of *CaERF67*, *CaERF73*, *CaERF127*, and *CaERF129*, which exhibited low expression in group IXb. The biological function of members of groups IXb and Xb might involve in capsorubin and capsaicinoids biosynthesis. Capsorubin and capsaicinoids are characteristically synthesized in pericarp and placental tissue, respectively. To further understand whether *CaERFs* are specifically expressed in different tissues, their expression patterns in the leaf, root, stem, pericarp and placenta were examined. The RNA-Seq raw data of leaves, roots and stems were not uploaded by Kim et al. [13]; the RPKM values were published instead, and when they were mapped to the *Capsicum annuum* genome (version 1.5), the expression of *CaERFs* clearly exhibited no tissue specificity (Fig. S6).

### Expression patterns of *CaERFs* in fruit pericarp and placenta in different developmental stages

**Fig. 5:**
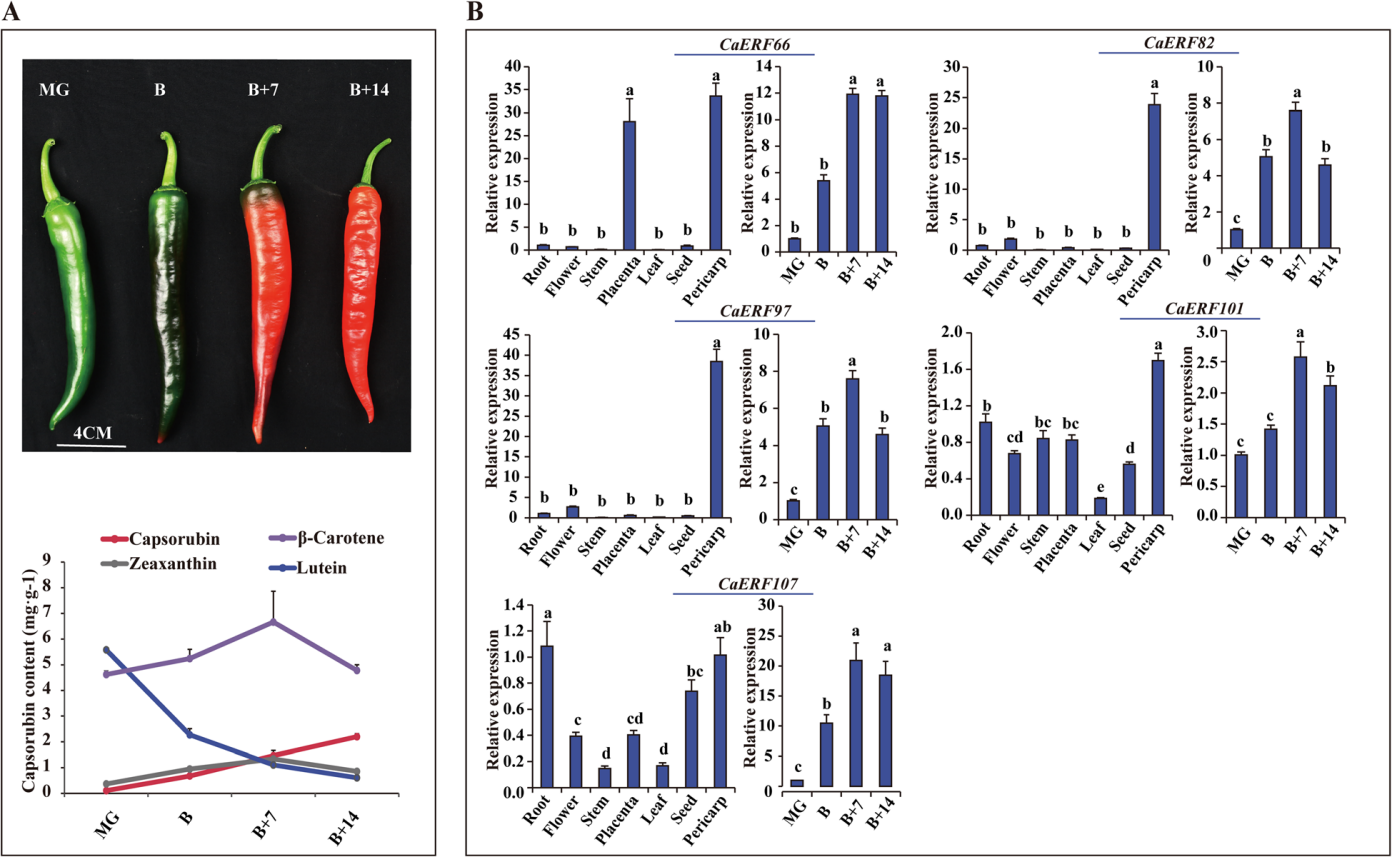
Expression patterns of five CaERF TFs in different tissues and developmental stages of the pericarp. **a** Phenotypes and carotenoids content (β-carotene, zeaxanthin. Lutein and capsorubin) in four developmental fruit stages, including mature green (MG), breaker (B), breaker plus 7 days (B + 7) and breaker plus 14 days (B + 14) stage. **b** Expression of 5 selected CaERF genes associated with carotenoids biosynthesis in the root, stem, level, flower, seed, placenta (16 DPA), pericarp (B) and four developmental pericarp stages. Different letters in the figures indicate significantly different values as determined by the analysis of three biological replicates (*P* < 0.05, Tukey’s test)

To further determine whether the expression of ERF genes possessed a specific stage in the pericarp and tissue, ten *CaERFs* from the clusters of possible candidates associated with capsorubin and capsaicinoids biosynthesis that are highly expressed in the pericarp and placenta at different developmental stages, were selected for analysis with perform qRT-PCR experiments. As shown in Fig. 5a, the contents of β-carotene, zeaxanthin and capsorubin started to increasingly accumulate in pericarp tissue at the MG stage, whereas lutein content including the branch of the non-synthetic capsorubin was decreased. The expression of *CaERF82, CaERF97, CaERF66, CaERF107* and *CaERF101* in pericarp tissue not only maintained a good agreement with the tendency of carotenoids biosynthesis (β-carotene, zeaxanthin and capsorubin), but also it exhibited a lower level of transcription in other tissue (roots, flowers, stems, placentas, leaves and seeds) (Fig. 5b). Thus, it was likely that the members of cluster C9 and cluster C10 were involved in carotenoids biosynthesis.

### Validation of capsaicinoids biosynthesis related ERF TFs

**Fig. 6:**
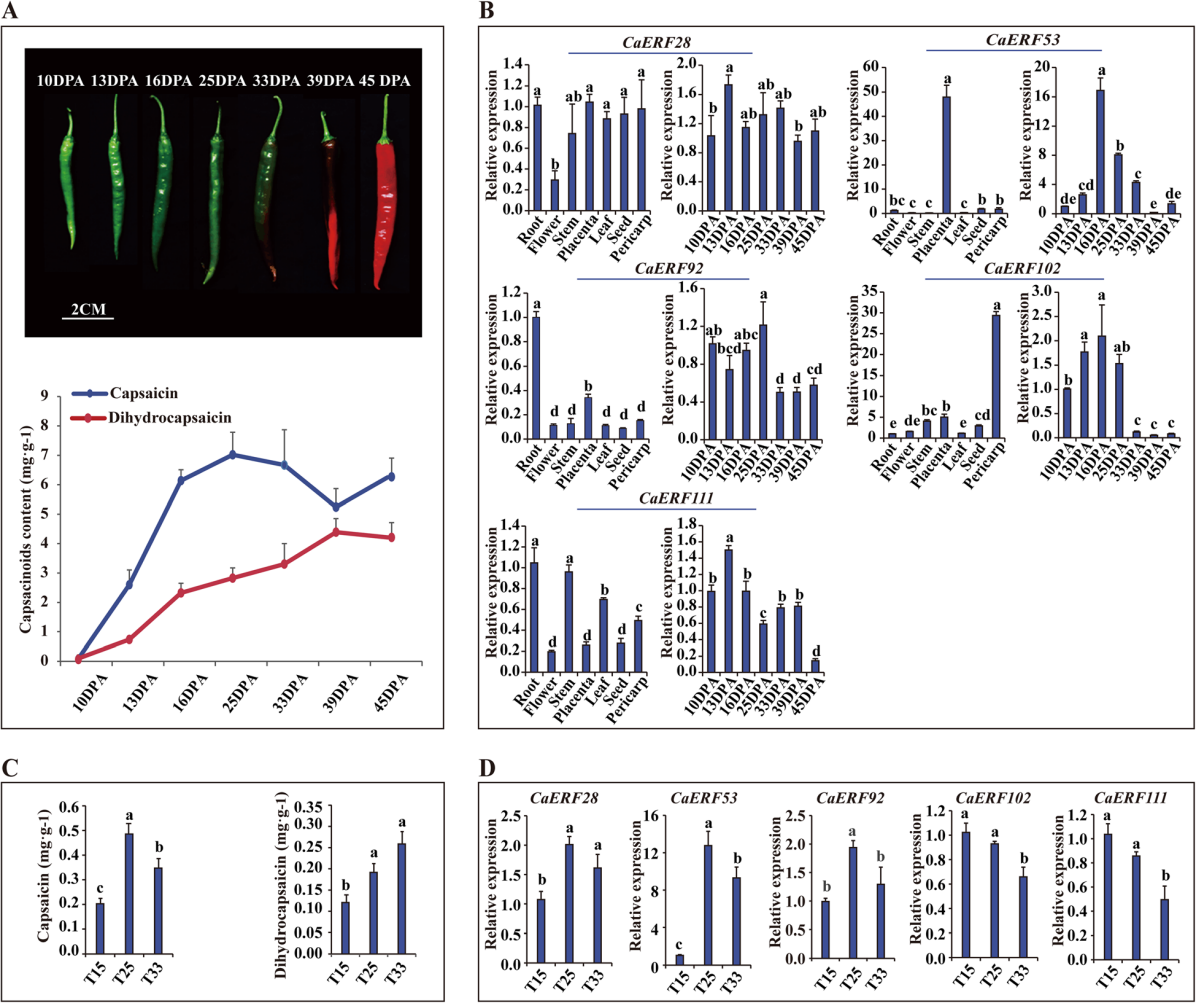
Expression patterns of five *CaERF* genes in different tissues and developmental stages of the placenta. **a** Phenotypes and capsaicinoid content (capsaicin and dihydrocapsaicin) during seven developmental stages of fruit as described previously [11]: 10, 13, 16, 25, 33, 39 and 45 DPA stages. **b** Expression of 5 selected *CaERFs* associated with capsaicinoids biosynthesis in the root, stem, level, flower, seed, placenta (16DPA), pericarp (16DPA) and in seven developmental pericarp stages. **c** The content of capsaicin and dihydrocapsaicin in response to different temperatures. D. The expression of *CaERF* genes involved in capsaicinoids biosynthesis in response to different temperatures. Different letters in the figures indicate significantly different values as determined analysis of three biological replicates (P < 0.05, Tukey’s test)

The capsaicin and dihydrocapsaicin content significantly increased in placental tissue from 10 DPA to 25 DPA, after which they increased slowly (Fig. 6a). The pattern of expression levels *CaERF102, CaERF53, CaERF111* and *CaERF92* in placental tissue were similar to the capsaicinoids biosynthesis patterns, while *CaERF28* expression did not show a developmental stage-regulated pattern. With the exception of *CaERF53*, these genes were also highly expressed in certain tissues (Fig. 6b). Additionally, we aimed to obtain a preliminary understanding of whether capsaicinoids biosynthesis was regulated by *CaERF* genes in pepper to enable adaption to different temperatures. As shown in Fig. 6c, the capsaicin and dihydrocapsaicin content dramatically accumulated with increasing temperature but the capsaicin content at T25 was significantly higher than it was in T33. The expression of *CaERF53, CaERF92* and *CaERF28* was the highest in T25, which was consistent with the accumulated level of capsaicin, while the expression of *CaERF102* and *CaERF111* decreased with increasing temperature (Fig. 6d). Therefore, these results indicated that *CaERF102, CaERF53, CaERF111* and *CaERF92* might be associated with capsaicinoids biosynthesis in pepper, but they perform different functions response to temperature to control capsaicinoids biosynthesis.

## Discussion

The AP2/ERF superfamily is one of the largest TF families in the plant kingdom, and it has been successfully identified and investigated in many plant species of sequenced genomes [35–37]. Although the AP2/ERF superfamily in peppers was reported by Jin et al. [38], they indicated that CaAP2/ERFs might be involved in the response to *P. capsici* in peppers. Capsorubin and capsaicinoids are unique to *Capsicum* spp., and they possess high economic and nutritional values. This study put more emphasis on demonstrating the relationship between *Capsicum*-specific secondary metabolites and the ERF family (the largest branch of the AP2/ERF superfamily). study of the *Capsicum* genome contributes to understanding the structure of gene families and predicting their biological functions. In this study, a total of 142 non-redundant *ERF* genes were identified from the *Capsicum annuum* genome. The ERF family in *Arabidopsis* (122) [15], watermelon (120) [36], rice (143) [39], Chinese cabbage (248) [40], cauliflower (146) [36] and *Bryum argenteum* (75) [41] were successfully identified and investigated. These results indicated that the number of *ERF* genes in different plants was distinct. Additionally, alignment analyses showed that the members of the ERF and DREB subfamilies possessed a specific WLG motif, as observed in the report of Cui et al. [37]. The distinction between the ERF and DREB subfamilies is that they can interact with the different motifs. The ERF subfamily typically binds to the GCC-box in the promoter regions, whereas the DREB subfamily is characterized by dehydration-responsive element binding factor containing a core motif of CCGAC [29, 42] According to Nakano and Sakuma’s study [15, 16], this DNA-binding specificity is mainly determined by the 14th and 19th amino acids in the AP2 domain (V14 and E19 for the DREB subfamily but A14 and D19 for the ERF subfamily); however, the DREB subfamily is completely conserved at V15 and E20, and the ERF subfamily is highly conserved at A14 and D19 (Fig. 1).

All CaERF members were used to construct a phylogenetic tree with matched proteins from tomato, rice and *Arabidopsis*. The classification of the tree was defined and annotated based on the proposed by Nakano et al. [15], and it ultimately defined 11 groups. This result was similar to that of Jin’s study in peppers [38], no matter the topology or classification of the tree. However, in this study, both groups X and IX were subdivided, and a new group XI was identified. Group XI showed a very low conservation of certain amino acids, which resulted in difficult classification. They group was classified as the ‘ERF-like subfamily’. It was likely that many gene signature motifs underwent divergent evolution after duplication from a common ancestor. Moreover, groups IXb and Xb were regarded as putative ‘pepper-specific groups’ (Fig. 2), and we cannot completely rule out the possibility that the members of putative “pepper-specific groups” were related to capsorubin and capsaicinoids biosynthesis. However, the members of these TFs were rarely expressed both in the pericarp and placenta throughout different developmental stages. Therefore, it seems that these ‘pepper-specific groups’ are not the master regulators of capsorubin and capsaicinoids biosynthesis.

Numerous studies have indicated that the members of a group in large families of plant TFs generally possess similar conserved amino acid motifs or domains, such as MYB, WAKY, and NAC [43–45]. In most cases, similar amino acid motifs are likely to share a similar function. Motifs 1 to 5, which are mainly located in the AP2 domain region were defined as “general motifs”, (Fig. 3b). Motifs 6 to 15 distributed outside the AP2 domain and were designated as “specific motifs” (Fig. 3b); they are potentially related to nuclear localization and transcription regulation [46]. Some reports suggested that the D(I/V) QAA sequences were regarded as the basic characteristics for the DREB family in cauliflower [36, 47], whereas motif 8 contained these conserved sequences, and it was primarily restricted to groups VI and X of the ERF family (Fig. 3). It was likely because TFs have occurred divergent evolution in different species. Indeed, groups VI and X in the phylogenetic tree were near the branch of the DREB family (Fig. 2).

In some cases, the same phylogenetic subgroup had a similar transcript level [48], implying that members of the same phylogenetic subgroup might perform similar functions. *SlERF6* was involved in the regulation of carotenoids biosynthesis and fruit ripening in tomato (Table S4) [18], which was located in group VII (Fig. S5). However, in this study, the genes of cluster C9 and cluster C10 were from different groups (except for CaERF101, which was in group VII), and they were regarded as candidates for the regulation of capsorubin biosynthesis. Because their expression patterns exhibited good agreement with the transcriptional level of the capsorubin synthesis gene (Fig. 4ab), and the members of this two pericarp highly expressed cluster (*CaERF82, CaERF97, CaERF66, CaERF107* and *CaERF101*) maintained good agreement with the increase in carotenoids biosynthesis (β-carotene, zeaxanthin and capsorubin) in pericarp tissue (Fig. 5b). These results indicated that the genes of the same phylogenetic subgroup exhibited distinct expression patterns, which is consistent with the observation from a previous study [48, 49]. Moreover, previous studies have demonstrated that *CaERF101* is involved in multiple secondary metabolic pathways and phytohormone, such as pungent capsaicinoids, polyamine and ABA biosynthesis [31, 50, 51]. Thus, it is likely that *CaERF101* also regulates secondary metabolic pathways in the ripening pericarp, and the members of cluster C9 and cluster C10 are involved in carotenoids biosynthesis.

*Erf* and *Jerf* in the pepper have been proposed to be involved in accumulation of pungency [31], and they were mapped to *CaERF53* and *CaERF101*, respectively, in this study. CaERF101 was identified as the putative orthologue of both CaPF1 and JERF1 in other reports, and it was shown to be associated with polyamine and ABA biosynthesis (Table S5) [50–52]. It is likely that the members of the group containing CaERF53 and CaERF101 (VII) regulates capsaicinoids or secondary metabolite biosynthesis. However, the members of cluster L3 (*CaERF102*, *CaERF85*, *CaERF101*, *CaERF65*, *CaERF73* and *CaERF116*) and cluster L4 (*CaERF111*, *CaERF92*, *CaERF28*, *CaERF53*, *CaERF103*, *CaERF114*, *CaERF25* and *CaERF139*) were candidates for the regulation of capsaicinoids biosynthesis in placental tissue, and only *CaERF53* came from group VII. However, *CaERF101* was placed into cluster L5. The expression of these members rapidly increased at 38 DPA, which was not similar to the stages of abundant-capsaicinoids accumulation (Fig. 4C and 5D). These results implied that *CaERF101* may perform multiple functions in addition to capsaicinoids biosynthesis. Moreover, the expression of four *CaERFs CaERF102, CaERF53, CaERF111* and *CaERF92* showed a positive correlation with the level of capsaicinoids biosynthesis (Fig. 6ab). In addition, capsaicinoids biosynthesis is regulated by environmental factors. ERF TF transcription is influenced by different temperatures, and ERF TFs have been shown to enhance plant tolerance to stress by being partially responsible for increasing certain metabolites [53, 54]. For example, overexpression of DREB1A can cause accumulation of monosaccharides, disaccharides, trisaccharides, and sugar alcohols to improve the tolerance to freezing and dehydration stress in transgenic plants [55]. In this study, the placenta significantly accumulated capsaicin and dihydrocapsaicin content following the higher temperature treatment. The expression of *CaERF53* and *CaERF92* increased, but that of *CaERF102* and *CaERF111* decreased with increasing temperature (Fig. 6d). Therefore, it may be that the members of cluster L3 and cluster L4 are related to temperature mediated capsaicinoids biosynthesis. However, *CaERF111*, *CaERF92, CaERF102* and *CaERF111* might play different roles in the regulation of capsaicinoids biosynthesis when exposed to different temperatures.

## Conclusion

a total of 142 members in the ERF family were identified in the pepper, and they were divided into DREB and ERF subfamilies. The DREB subfamily is completely conserved at V15 and E20, while the ERF subfamily is highly conserved at A14 and D19. The phylogenetic analysis of the ERF family resulted in a distribution of 11 groups, of which the DREB subfamily included group I to group IV, and the ERF subfamily contained group V to group XI. Generally, the same group of trees possessed similar motif patterns. Motifs 1 to 5 are present in the largest number of CaERF proteins and were thus designated “general motifs”, whereas other motifs distributed outside the AP2 domain were classified as “specific motifs”. The members of cluster C9 and cluster C10 might be involved in capsorubin biosynthesis, especially those with high expression: *CaERF82, CaERF97, CaERF66, CaERF107* and *CaERF101*. These five genes not only showed a trend that was similar to that of the accumulation of carotenoids biosynthesis genes (β-carotene, zeaxanthin and capsorubin) in pericarp tissue, but also were expressed at low levels in other tissues. The genes in cluster L3 and cluster L4 were likely associated with the regulation of capsaicinoids biosynthesis. *CaERF102, CaERF53, CaERF111* and *CaERF92*, which were identified in cluster L3 and cluster L4, maintained good expression pattern consistency with the accumulated level of capsaicinoids. In response to different temperatures, these ERF TFs may have different roles in mediating capsaicinoids biosynthesis. However, whether these candidate ERF TFs exert control over pepper-specific metabolite biosynthesis requires further study.

## Methods

### Identification of ERFs in the pepper genome

*ERF* genes were retrieved from the latest version, 2.0 of the *Capsicum annuum* genome. Which is available in the Pepper Genome Platform (http://peppergenome.snu.ac.kr/), by using the Hidden Markov Model (HMM) profile of the AP2 domain (PF00847) from the PFAM database with a predefined threshold of e-value <1e^− 5^. Redundancy sequences, genes containing two or more AP2 domains, and those with one AP2 domain coexisting whit the B3 domain were filtered by HMMER (http://hmmer.org/) and NCBI Conserved Domain Search Service (CD Search) (https://www.ncbi.nlm.nih.gov/Structure/bwrpsb/bwrpsb.cgi). The full-length amino acid sequences (length), theoretical isoelectric point (PI), molecular weight (MW) and instability index of ERF proteins were predicted by using the compute pI/Mw tool in the ExPASy server (http://www.expasy.org/).

### Multiple sequence alignment and phylogenetic analysis

All of the ERF amino acid sequences were aligned using Clustal X 2.1 (http://www.clustal.org/) with the default parameters. Unrooted neighbour joining (NJ) trees were constructed with 1000 bootstrap replications using MEGA X (https://www.megasoftware.net/) [56]. For the generation of tree from three additional species, the ERF proteins of rice (138) and tomato (137) were obtained from Plant TF Database version 4.0 (http://planttfdb.cbi.pku.edu.cn/download.php) [57], and those of *Arabidopsis* were obtained from Nakano et al. (Table S1) [15]. The trees were constructed and visualized using Evolview (http://www.evolgenius.info/evolview).

### Analysis of conserved motifs

Functional motifs or domains were identified via MEME (http://meme.nbcr.net/meme/cgi-bin/meme.cgi) using the following parameters: site distribution, any number of repetitions; number of motifs, 15; minimum motif width, 6; maximum motif width, 100; the minimum number of sites, 5; and maximum number of sites: 100 [58]. The motifs were constructed and visualized using Dual Systeny Plotter software (https://github.com/CJ-Chen/TBtools).

### RNA-seq data analysis

The pepper RNA-seq data (GenBank: AYRZ01000000) were downloaded from the SRA database (http://www.ncbi.nlm.nih.gov/sra). The fragments per kilobase of exon per million fragments mapped (FPKM) values were obtained from all candidate CaERFs with Cufflinks and TopHat based on the *Capsicum annuum* genome version 2.0 [59, 60]. Seven RNA-seq data of different placenta-developmental stages at day-post-anthesis (DPA) were analysed, 6, 16, 25, 36, 38, 43 and 48 DPA. Heat maps showing the expression patterns of genes were constructed with the R Programming Language (R) software (https://www.r-project.org/).

### Plant materials

Fifty-nine inbred line (*Capsicum annuum*) seeds, which is preserved by our lab [11], were sown in a plug tray with mixture substrate (peat, coir pith and perlite). When seedlings grew to the five-leaf stage, the root, stem and leaf were collected, immediately frozen in liquid nitrogen and stored at − 80 °C, some of the plants were cultivated in greenhouse conditions with a mixture substrate and were fertilized every week with water-soluble fertilizer (N: P: K, 20:20:20, Plant-Soul, China). Flowers underwent artificial pollination at zero DPA, and the flowers were only left in the fifth node. The placentas of 10, 13, 16, 25, 33, 39 and 45 DPA fruits were collected for RNA extraction and the analysis of capsaicinoids content. Moreover, the mature green stage (MG) occurred at 30 DPA, the breaker (B) was marked by darkening of the pericarps colour and the first appearance of red colour, and the following stages were breaker plus 7 days (B + 7) and breaker plus 14 days (B + 14). The four stages of pericarps were collected, frozen in liquid nitrogen and stored at − 80 °C until the RNA was extract RNA and examine carotenoids content.

### Temperature treatments

Peppers (*Capsicum annuum* L. cv. No. 59) were cultivated in greenhouse conditions. Flowers in the fifth node underwent artificial pollination at zero DPA. For the temperature treatment, when flower buds in the fifth node began to bloom, the plants were transferred to a growth chamber under the following growth conditions: 60–70% humidity; 350 μmol·m^− 2^·s^− 1^ light intensity; and 12 h/12 h (light/dark cycle). A total of 3 temperature treatments were performed: 15 °C (T15), 25 °C (T25), and 33 °C (T33). The placentas from 16 DPA fruits were collected for RNA extraction. The placentas from 45 DPA fruits were collected and oven-dried at 75 °C for 48 h, and then they were stored at − 20 °C until capsaicinoids content was examine.

### RNA extraction and quantitative real-time PCR (qPCR)

Total RNA was isolated from placentas using a Magen HiPure Total RNA Mini kit (R4111, Magen, China) according to the manufacturer’s instructions. First-strand cDNA synthesis was performed with approximately 500 ng of RNA using a HiScript II 1st Strand cDNA Synthesis kit (R211–01, Vazyme, China) in a reaction volume of 20 μL. The synthesized cDNA was diluted 10 times with sterile water, and then templates were used in qPCR. Primers were designed based on Primer 5.0 software for qPCR. All primer sequences are listed in Table S2.

The qPCRs were carried out in a Bio-Rad CFX384 Touch TM system with qPCR SYBR Green Master Mix (Q131–02, Vazyme, China). The reaction mix was 1 μL of cDNA template, 0.2 μL of each primer (10 μmol/μL), 5 μL of SYBR Green Master Mix, and 3.6 μL of nuclease-free water. The PCR amplification conditions were as follows: 95 °C for 5 min; then 40 cycles at 95 °C for 5 s and 60 °C for 30 s. A melting-curve analysis was performed at 95 °C for 5 s, which was followed by a temperature increase from 60 °C to 95 °C. Additionally, CA00g52149 and CA12g20490 (ID in version 1.55 of the *Capsicum annuum* genome) were used as housekeeping genes; they were identified in the pepper genome and the data were unpublished. The relative expression of each ERF gene was calculated with the 2^−ΔΔCt^ method [61]. The qPCRs using the placenta were performed with biological triplicates. The results were analysed statistically using SPSS 22 with Dunnett’s *t*-test to determine significant differences.

### Quantification of carotenoids and capsaicinoids content

Oven-dried placental tissue from pepper fruits was ground into fine powder with a mortar and pestle. A total of 0.1 g that was extracted from the samples was mixed with 5 ml of methyl alcohol and tetrahydrofuran (1:1, HPLC grade) in 15 ml of centrifuge tubes, and then they were ultrasonicated for 30 min. These samples were extracted for 12 h at room temperature.1 millilitre of the supernatant was collected and filtered through a 0.22 μm millipore membrane, and then the capsaicinoids content was determined by an HPLC system (Alliance E2695, Waters, America).

Frozen pericarp tissues were ground by a mortar and pestle, and then freeze-dried for 24 h with a freeze dryer (Labconco/Freezone, Labconco, America). A total of 0.5 g of the freeze-dried samples were added to 50 ml centrifuge tubes with 8 ml of extracting solution containing hexyl hydride, acetone and absolute ethyl alcohol (2:1:1, HPLC grade), and then the samples underwent ultrasonic-assisted extraction for 30 min. Five millilitres of the supernatants was transferred into 50 ml centrifuge tubes and were mixed with 5 ml of extraction solution. Then, the mixtures were mixed with an equal proportion of NaCl saturated solution (100%). The supernatants and 2 ml of KOH and methyl alcohol (1:9) were mixed and incubated for 12 h at room temperature. Finally, the extract was mixed 2 ml of MTBE and NaCl saturated solution (100%). The supernatant was rinsed three times using a NaCl saturated solution (100%). One millilitre of the supernatant was used to determine carotenoids content by an HPLC system (Alliance E2695, Waters, America).

## Supplementary information

**Additional file 1: Fig. S1.** Carotenoids (A) and capsaicinoids (B) biosynthetic pathways. **Fig. S2.** Physical distribution of all candidate *CaERFs* among chromosomes. **Fig. S3.** Multiple sequence alignment of the DREB and ERF protein subfamilies. **Fig. S4.** Phylogenetic tree of the pepper ERF family in relation to *Arabidopsis.* **Fig. S5.** Phylogenetic tree of the pepper ERF family in relation to tomato (137), rice (138) and *Arabidopsis* (122). **Fig. S6.** The expression patterns of *CaERF* genes in different tissues.

**Additional file 2: Table S1.** *ERF* family genes in *Arabidopsis*, tomato and rice. **Table S2.** List of primers used in real-time quantitative PCR (qPCR). **Table S3.** *CaERF* genes identified and characterized in the pepper. **Table S4.** Biological functions of characterized ERF proteins that potentially exist in *Arabidopsis* and tomato. **Table S5.** Putative CaERF homologs (version 2.0) of pepper ERF proteins with known biological functions. **Table S6.** Multilevel consensus sequence identified in 144 *CaERF* genes.

## Data Availability

All data generated or analyzed during this study are included in this article and its additional files.

## Abbreviations

TFs: Transcription factors
Chr: Chromosome
FPKM: Fragments Per Kilobase of transcript per Million mapped reads
AP2/ERF: Apetala2/ Ethylene response factor ERF
DREB: Dehydration responsive element binding proteins
GGPP: Geranylgeranyl pyrophosphate
PSY: Phytoene synthase
PDS: Phytoene desaturase
LCYB: Lycopene β-cyclase
Pal: Phe ammonia-lyase
Comt: Caffeic acid O-methyltransferase
AT3: A putative acyltransferase
CCS: Capsanthin/capsorubin synthase
CS: Cpsaicin synthase
ZDS: ζ-carotene desaturase
CRTISO: Carotenoids isomerase
LCYE: Lycopene ε-cyclase
CrtZ-2: β-carotene hydroxylase-2
PAL: Phenylalanine ammonia-lyase
C4H: Cinnamate 4-hydroxylase
4CL: 4-coumaroyl-CoA ligase
HCT: Hydroxycinnamoyl transferase
C3H: p-coumaroyl shikimate/quinate 3-hydroxylase
COMT: Caffeoyl-CoA 3-O-methyltransferase
HCHL: Hydroxycinnamoyl-CoA hydratase lyase
AMT: Aminotransferase
BCAT: Branched-chain amino acid aminotransferase
Kas: Ketoacyl-ACP synthase
ACL: Acyl carrier protein
FatA: Acyl-ACP thioesterase
HMM: Hidden markov model
DPA: Day-post-anthesis
MG: Mature green stage B: Breaker
B + 7: Breaker plus 7 days
B + 14: Breaker plus 14 days
