# Supplementary material for: Systematic analysis of the Capsicum ERF transcription factor family: identification of regulatory factors involved in the regulation of species-specific metabolites
Source: BMC Genomics. 2020 Aug 24;21:573. doi: 10.1186/s12864-020-06983-3 (PMC7444197; doi:10.1186/s12864-020-06983-3)
Supplement: Supplementary file 1 — Additional file 1: Fig. S1. Carotenoids (A) and capsaicinoids (B) biosynthetic pathways. Fig. S2. Physical distribution of all candidate CaERFs among chromosomes. Fig. S3. Multiple sequence alignment of the DREB and ERF protein subfamilies. Fig. S4. Phylogenetic tree of the pepper ERF family in relation to Arabidopsis. Fig. S5. Phylogenetic tree of the pepper ERF family in relation to tomato (137), rice (138) and Arabidopsis (122). Fig. S6. The expression patterns of CaERF genes in different tissues. [file 12864_2020_6983_MOESM1_ESM.pdf]

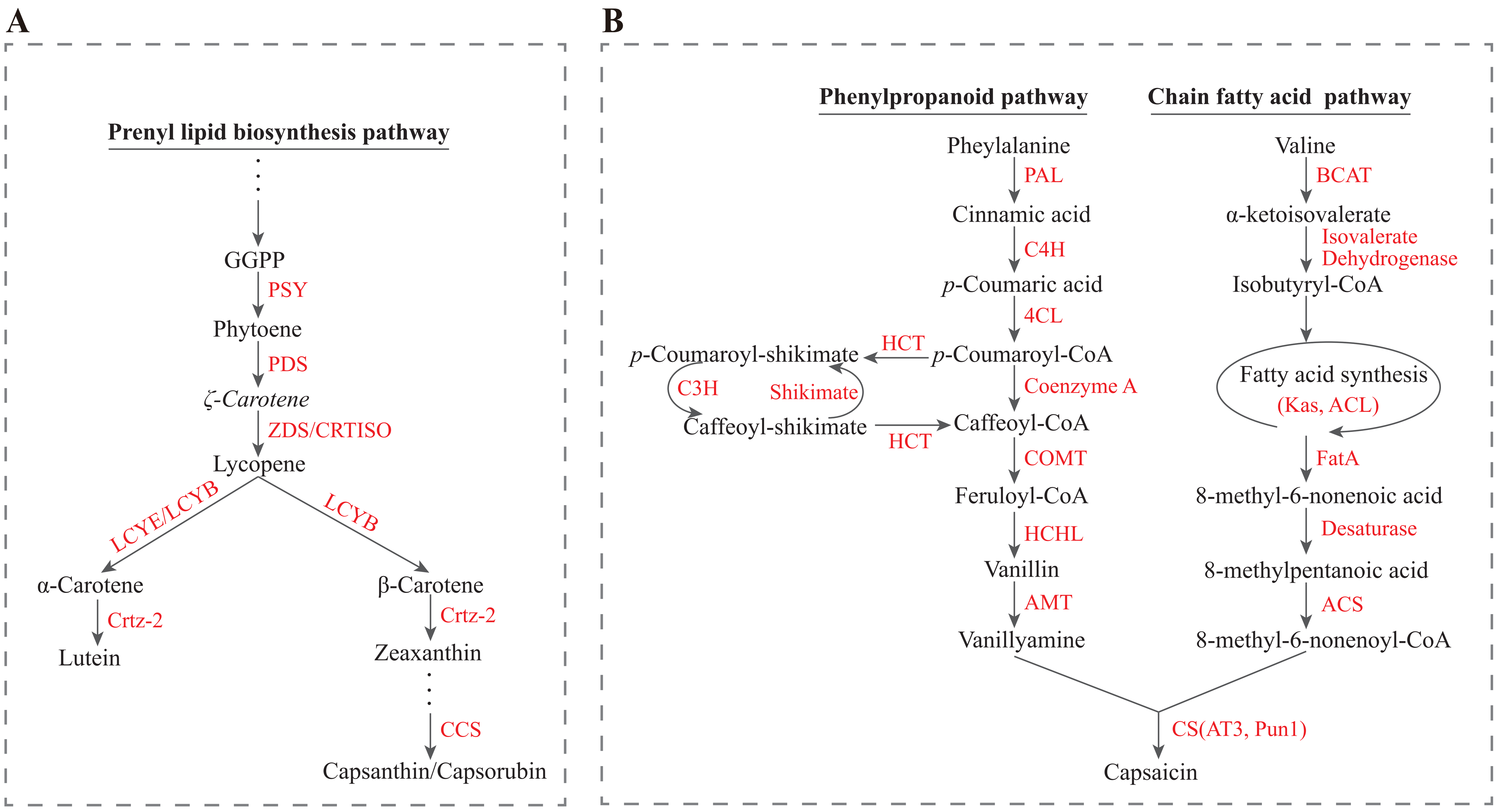

Fig. S1. Carotenoids (A) and capsaicinoids (B) biosynthetic pathways. A. Geranylgeranyl diphosphate that is synthesized from the prenyl lipid biosynthesis pathway is catalyzed by phytoene synthase to produce phytoene, which represents the first step in the carotenoid pathway. Then, after synthesizing lycopene, this pathway is divided into two different branches: α-carotene is finally developed into lutein, and β-carotene is ultimately formed into capsorubin or capsanthin as a function of capsanthin/capsorubin synthase (CCS). The ellipsis indicates intermediate steps. B. The two distinct pathways, the phenylalanine and chain fatty acid biosynthesis pathways, come together to produce capsaicin at the end of the catalysis of capsaicin synthase (CS). ZDS, ζ-carotene desaturase; CRTISO, carotenoids isomerase; LCYE, lycopene ε-cyclase; CrtZ-2, β-carotene hydroxylase-2; PAL, phenylalanine ammonia-lyase; C4H, cinnamate 4-hydroxylase; 4CL, 4-coumaroyl-CoA ligase; HCT, hydroxycinnamoyl transferase; C3H, p-coumaroyl shikimate/quinate 3-hydroxylase; COMT, caffeoyl-CoA 3-O-methyltransferase; HCHL, hydroxycinnamoyl-CoA hydratase lyase; AMT, aminotransferase; BCAT, branched-chain amino acid aminotransferase; Kas, ketoacyl-ACP synthase; ACL, acyl carrier protein; and FatA, acyl-ACP thioesterase.

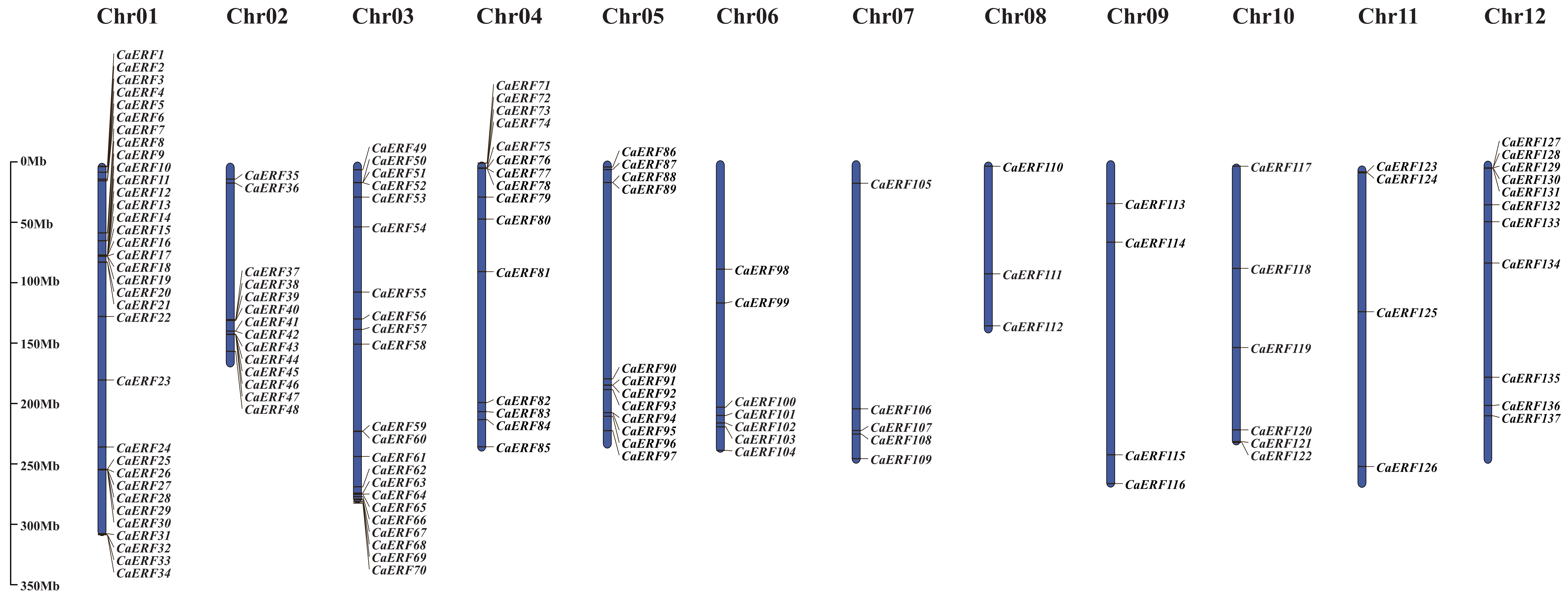

Fig. S2. Physical distribution of all candidate *CaERFs* among chromosomes (Chrs). The chromosome distribution of each pepper *ERF* was obtained from the genome annotation information, and then used the Map Gene 2 Chromosome v 2 (MG2C; http://mg2c.iask.in/mg2c_v2.0/) to make the linkage visualization.

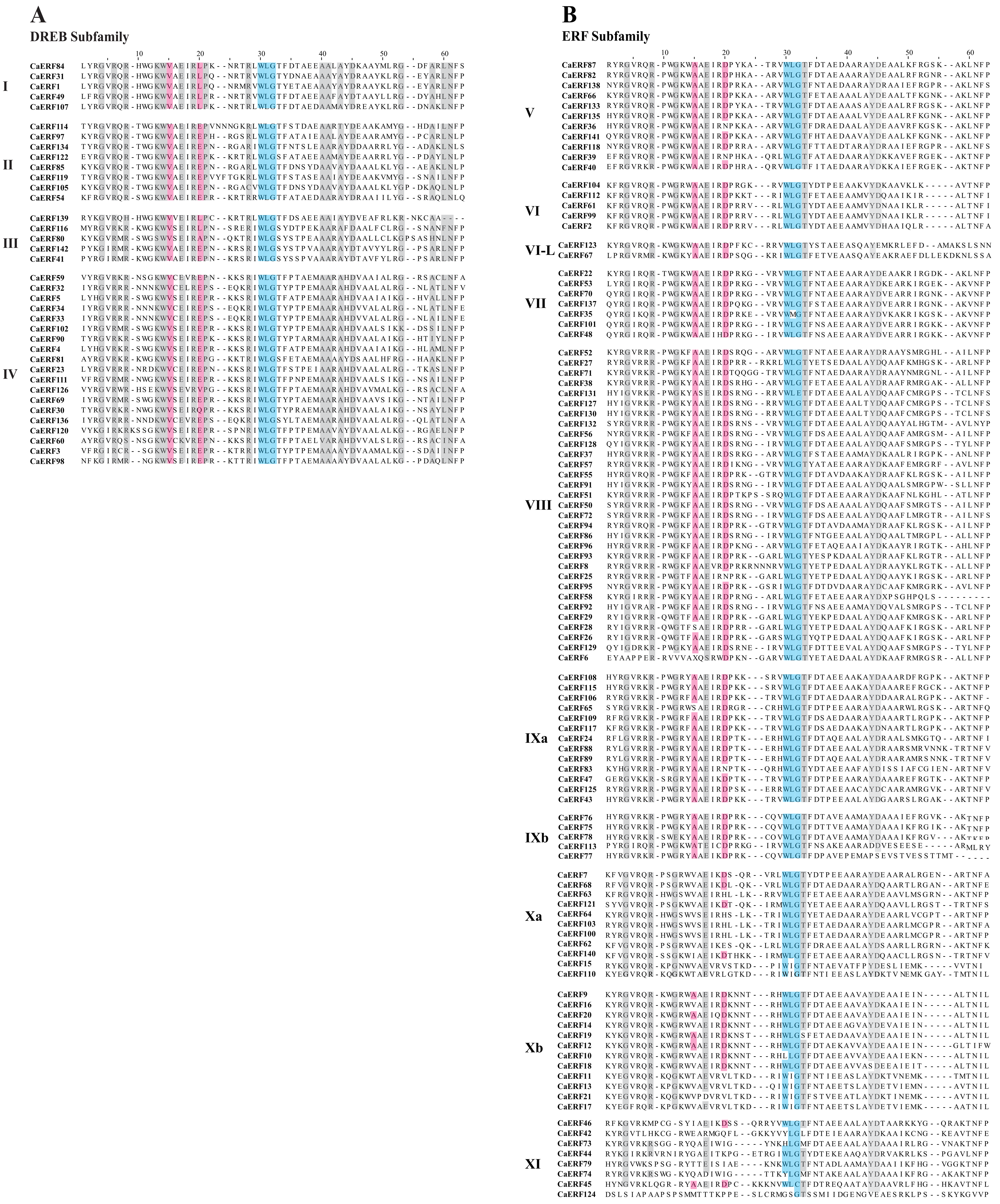

Fig. S3. Multiple sequence alignment of the DREB and ERF protein subfamilies. The amino acid sequences of these proteins were aligned by Clustal W. The red color indicated characteristic amino acid in the DREB and ERF subfamily, respectively. The blue color indicated the specific WLG motif. The gray color indicated more than 90% of highly conserved residues in the two subfamilies.

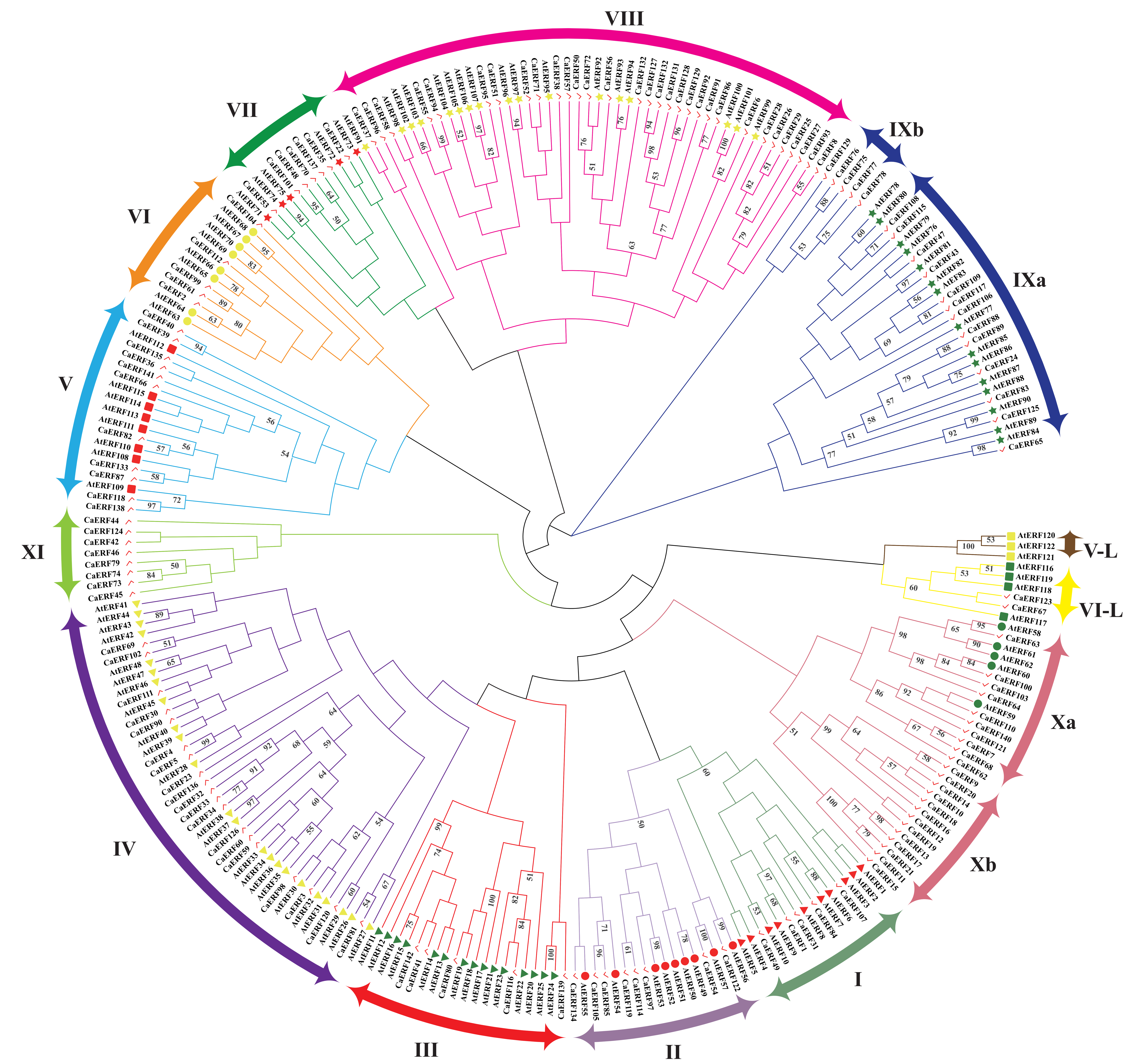

Fig. S4. Phylogenetic tree of the pepper ERF family in relation to Arabidopsis. The different-colored branch indicate different groups of ERF proteins. Red check represented ERF proteins in pepper, other different olor shapes indicated different groups in phylogeny of *Arabidopsis* (Nakano et al., 2006).

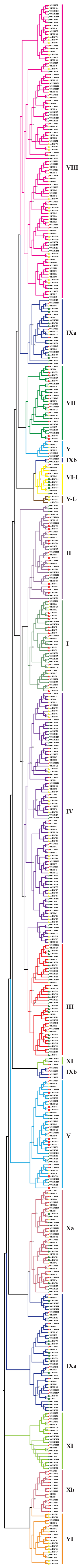

Fig. S5. Phylogenetic tree of the pepper ERF family in relation to tomato (137), rice (138) and *Arabidopsis* (122). The different-colored branch indicate different groups of ERF proteins. Red, yellow and green check represented ERF proteins in pepper, tomato and rice, respectively. Other different olor shapes indicated different groups in phylogeny of *Arabidopsis* (Nakano et al., 2006).

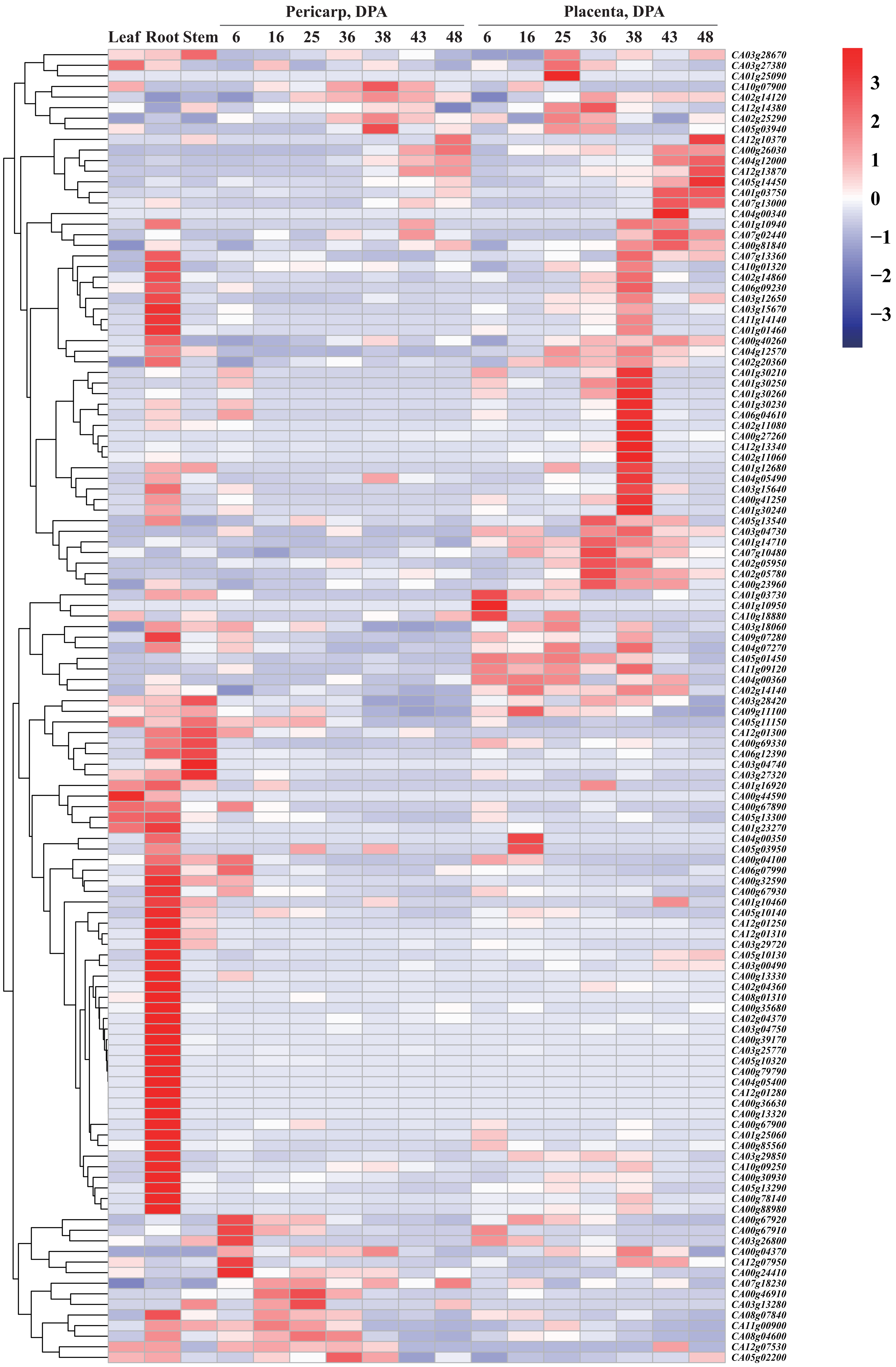

Fig. S6. The expression patterns of *CaERF* genes in different tissues . The heat map was directly constructed by using the RPKM value. The RPKM value was mapped to the *Capsicum annuum* genome of version 1.5 performed by Kim et al (2014).
