# Supplementary material for: Systematic analysis of the Capsicum ERF transcription factor family: identification of regulatory factors involved in the regulation of species-specific metabolites
Source: BMC Genomics. 2020 Aug 24;21:573. doi: 10.1186/s12864-020-06983-3 (PMC7444197; doi:10.1186/s12864-020-06983-3)
Supplement: Supplementary file 2 — Additional file 2: Table S1. ERF family genes in Arabidopsis, tomato and rice. Table S2. List of primers used in real-time quantitative PCR (qPCR). Table S3. CaERF genes identified and characterized in the pepper. Table S4. Biological functions of characterized ERF proteins that potentially exist in Arabidopsis and tomato. Table S5. Putative CaERF homologs (version 2.0) of pepper ERF proteins with known biological functions. Table S6. Multilevel consensus sequence identified in 144 CaERF genes. [file 12864_2020_6983_MOESM2_ESM.docx]

**Table S1.** *ERF* family genes in *Arabidopsis*, tomato and rice.

| **Rice** | | | **Tomato** | | ***Arabidopsis*** | |
| --- | --- | --- | --- | --- | --- | --- |
| **ID** | **Gene name** | | **ID** | **Gene name** | **ID** | **Gene name** |
| LOCOs01g04020.1 | *OsERF1* | Solyc01g005630.1.1 | | *SlERF1* | At2g20880 | *AtERF1* |
| LOCOs01g07120.1 | *OsERF2* | Solyc01g008880.1.1 | | *SlERF2* | At4g28140 | *AtERF2* |
| LOCOs01g10370.1 | *OsERF3* | Solyc01g009440.1.1 | | *SlERF3* | At1g36060 | *AtERF3* |
| LOCOs01g12440.1 | *OsERF4* | Solyc01g014720.1.1 | | *SlERF4* | At2g22200 | *AtERF4* |
| LOCOs01g21120.1 | *OsERF5* | Solyc01g057080.1.1 | | *SlERF5* | At5g65130 | *AtERF5* |
| LOCOs01g46870.1 | *OsERF6* | Solyc01g065980.2.1 | | *SlERF6* | At1g22190 | *AtERF6* |
| LOCOs01g54890.1 | *OsERF7* | Solyc01g067540.1.1 | | *SlERF7* | At1g78080 | *AtERF7* |
| LOCOs01g58420.1 | *OsERF8* | Solyc01g090300.2.1 | | *SlERF8* | At4g39780 | *AtERF8* |
| LOCOs01g59780.1 | *OsERF9* | Solyc01g090310.2.1 | | *SlERF9* | At1g64380 | *AtERF9* |
| LOCOs01g64790.1 | *OsERF10* | Solyc01g090320.2.1 | | *SlERF10* | At4g13620 | *AtERF10* |
| LOCOs01g66270.1 | *OsERF11* | Solyc01g090340.2.1 | | *SlERF11* | \| At1g46768 \| \| --- \| | *AtERF11* |
| LOCOs01g73770.1 | *OsERF12* | Solyc01g090370.1.1 | | *SlERF12* | At4g06746 | *AtERF12* |
| LOCOs02g06330.1 | *OsERF13* | Solyc01g090560.2.1 | | *SlERF13* | At2g23340 | *AtERF13* |
| LOCOs02g09650.1 | *OsERF14* | Solyc01g091760.2.1 | | *SlERF14* | At4g36900 | *AtERF14* |
| LOCOs02g10760.1 | *OsERF15* | Solyc01g095500.2.1 | | *SlERF15* | At5g67190 | *AtERF15* |
| LOCOs02g13710.1 | *OsERF16* | Solyc01g108240.2.1 | | *SlERF16* | At3g50260 | *AtERF16* |
| LOCOs02g29550.1 | *OsERF17* | Solyc02g067020.1.1 | | *SlERF17* | At1g21910 | *AtERF17* |
| LOCOs02g32040.1 | *OsERF18* | Solyc02g077360.1.1 | | *SlERF18* | At1g77640 | *AtERF18* |
| LOCOs02g32140.1 | *OsERF19* | Solyc02g077370.1.1 | | *SlERF19* | At1g44830 | *AtERF19* |
| LOCOs02g34260.1 | *OsERF20* | Solyc02g077810.1.1 | | *SlERF20* | At4g31060 | *AtERF20* |
| LOCOs02g34270.1 | *OsERF21* | Solyc02g077840.1.1 | | *SlERF21* | At5g21960 | *AtERF21* |
| LOCOs02g35240.1 | *OsERF22* | Solyc02g090770.1.1 | | *SlERF22* | At1g19210 | *AtERF22* |
| LOCOs02g38090.1 | *OsERF23* | Solyc02g090790.1.1 | | *SlERF23* | At1g74930 | *AtERF23* |
| LOCOs02g42585.1 | *OsERF24* | Solyc02g090800.1.1 | | *SlERF24* | At1g22810 | *AtERF24* |
| LOCOs02g43790.1 | *OsERF25* | Solyc02g093130.1.1 | | *SlERF25* | At1g71520 | *AtERF25* |
| LOCOs02g43820.1 | *OsERF26* | Solyc03g005500.1.1 | | *SlERF26* | At1g71450 | *AtERF26* |
| LOCOs02g43940.1 | *OsERF27* | Solyc03g005510.1.1 | | *SlERF27* | At1g33760 | *AtERF27* |
| LOCOs02g43970.1 | *OsERF28* | Solyc03g005520.1.1 | | *SlERF28* | At1g01250 | *AtERF28* |
| LOCOs02g45420.1 | *OsERF29* | Solyc03g006320.1.1 | | *SlERF29* | At2g36450 | *AtERF29* |
| LOCOs02g45450.1 | *OsERF30* | Solyc03g007460.1.1 | | *SlERF30* | At5g52020 | *AtERF30* |
| LOCOs02g51670.1 | *OsERF31* | Solyc03g026270.1.1 | | *SlERF31* | At1g63040 | *AtERF31* |
| LOCOs02g52670.1 | *OsERF32* | Solyc03g026280.2.1 | | *SlERF32* | At1g12630 | *AtERF32* |
| LOCOs02g54050.1 | *OsERF33* | Solyc03g093530.1.1 | | *SlERF33* | At5g51990 | *AtERF33* |
| LOCOs02g54160.1 | *OsERF34* | Solyc03g093540.1.1 | | *SlERF34* | At4g25490 | *AtERF34* |
| LOCOs02g55380.1 | *OsERF35* | Solyc03g093550.1.1 | | *SlERF35* | At4g25470 | *AtERF35* |
| LOCOs03g05590.1 | *OsERF36* | Solyc03g093560.1.1 | | *SlERF36* | At4g25480 | *AtERF36* |
| LOCOs03g07830.1 | *OsERF37* | Solyc03g093610.1.1 | | *SlERF37* | At1g63030 | *AtERF37* |
| LOCOs03g07940.1 | *OsERF38* | Solyc03g114440.1.1 | | *SlERF38* | At1g12610 | *AtERF38* |
| LOCOs03g08460.1 | *OsERF39* | Solyc03g116610.2.1 | | *SlERF39* | At2g44940 | *AtERF39* |
| LOCOs03g08470.1 | *OsERF40* | Solyc03g117130.2.1 | | *SlERF40* | At3g60490 | *AtERF40* |
| LOCOs03g08490.1 | *OsERF41* | Solyc03g117230.1.1 | | *SlERF41* | At3g16280 | *AtERF41* |
| LOCOs03g08500.2 | *OsERF42* | Solyc03g118190.2.1 | | *SlERF42* | At1g77200 | *AtERF42* |
| LOCOs03g09170.1 | *OsERF43* | Solyc03g119580.1.1 | | *SlERF43* | At2g35700 | *AtERF43* |
| LOCOs03g15660.1 | *OsERF44* | Solyc03g119800.1.1 | | *SlERF44* | At4g16750 | *AtERF44* |
| LOCOs03g22170.1 | *OsERF45* | Solyc03g120840.1.1 | | *SlERF45* | At5g25810 | *AtERF45* |
| LOCOs03g60120.1 | *OsERF46* | Solyc03g123500.2.1 | | *SlERF46* | At5g11590 | *AtERF46* |
| LOCOs03g64260.1 | *OsERF47* | Solyc03g124110.1.1 | | *SlERF47* | At2g25820 | *AtERF47* |
| LOCOs04g18650.1 | *OsERF48* | Solyc04g007180.1.1 | | *SlERF48* | At4g32800 | *AtERF48* |
| LOCOs04g32620.1 | *OsERF49* | Solyc04g012050.2.1 | | *SlERF49* | At3g11020 | *AtERF49* |
| LOCOs04g32790.1 | *OsERF50* | Solyc04g014530.1.1 | | *SlERF50* | At5g05410 | *AtERF50* |
| LOCOs04g34970.1 | *OsERF51* | Solyc04g050750.1.1 | | *SlERF51* | At2g38340 | *AtERF51* |
| LOCOs04g44670.1 | *OsERF52* | Solyc04g051360.2.1 | | *SlERF52* | At2g40350 | *AtERF52* |
| LOCOs04g46220.1 | *OsERF53* | Solyc04g054910.2.1 | | *SlERF53* | At2g40340 | *AtERF53* |
| LOCOs04g46240.1 | *OsERF54* | Solyc04g071770.2.1 | | *SlERF54* | At1g75490 | *AtERF54* |
| LOCOs04g46250.1 | *OsERF55* | Solyc04g072300.1.1 | | *SlERF55* | At5g18450 | *AtERF55* |
| LOCOs04g46400.1 | *OsERF56* | Solyc04g072900.1.1 | | *SlERF56* | At3g57600 | *AtERF56* |
| LOCOs04g46410.1 | *OsERF57* | Solyc04g078640.1.1 | | *SlERF57* | At2g40220 | *AtERF57* |
| LOCOs04g46440.1 | *OsERF58* | Solyc04g080910.1.1 | | *SlERF58* | At1g15360 | *AtERF58* |
| LOCOs04g48350.1 | *OsERF59* | Solyc05g009250.1.1 | | *SlERF59* | At5g19790 | *AtERF59* |
| LOCOs04g52090.1 | *OsERF60* | Solyc05g009450.1.1 | | *SlERF60* | At5g25190 | *AtERF60* |
| LOCOs04g55520.1 | *OsERF61* | Solyc05g013540.1.1 | | *SlERF61* | At5g11190 | *AtERF61* |
| LOCOs04g56150.1 | *OsERF62* | Solyc05g050790.1.1 | | *SlERF62* | At5g25390.1 | *AtERF62* |
| LOCOs04g57340.1 | *OsERF63* | Solyc05g050830.1.1 | | *SlERF63* | At4g11140 | *AtERF63* |
| LOCOs05g25260.1 | *OsERF64* | Solyc05g051180.1.1 | | *SlERF64* | At4g23750 | *AtERF64* |
| LOCOs05g27930.1 | *OsERF65* | Solyc05g051200.1.1 | | *SlERF65* | At5g53290 | *AtERF65* |
| LOCOs05g28350.1 | *OsERF66* | Solyc05g052030.1.1 | | *SlERF66* | At4g27950 | *AtERF66* |
| LOCOs05g29810.1 | *OsERF67* | Solyc05g052040.1.1 | | *SlERF67* | At3g61630 | *AtERF67* |
| LOCOs05g34730.1 | *OsERF68* | Solyc05g052050.1.1 | | *SlERF68* | At2g46310 | *AtERF68* |
| LOCOs05g36100.1 | *OsERF69* | Solyc05g052410.1.1 | | *SlERF69* | At1g22985 | *AtERF69* |
| LOCOs05g37640.1 | *OsERF70* | Solyc06g035700.1.1 | | *SlERF70* | At1g71130 | *AtERF70* |
| LOCOs05g39590.1 | *OsERF71* | Solyc06g050520.1.1 | | *SlERF71* | At2g47520 | *AtERF71* |
| LOCOs05g41760.1 | *OsERF72* | Solyc06g051840.1.1 | | *SlERF72* | At3g16770 | *AtERF72* |
| LOCOs05g41780.1 | *OsERF73* | Solyc06g053240.2.1 | | *SlERF73* | At1g72360 | *AtERF73* |
| LOCOs05g49010.1 | *OsERF74* | Solyc06g054630.1.1 | | *SlERF74* | At1g53910 | *AtERF74* |
| LOCOs05g49700.1 | *OsERF75* | Solyc06g063070.2.1 | | *SlERF75* | At3g14230.1 | *AtERF75* |
| LOCOs06g03670.1 | *OsERF76* | Solyc06g065820.2.1 | | *SlERF76* | At1g28370 | *AtERF76* |
| LOCOs06g06540.1 | *OsERF77* | Solyc06g066540.1.1 | | *SlERF77* | At1g03800 | *AtERF77* |
| LOCOs06g06970.1 | *OsERF78* | Solyc06g068360.2.1 | | *SlERF78* | At3g15210 | *AtERF78* |
| LOCOs06g07030.1 | *OsERF79* | Solyc06g068830.1.1 | | *SlERF79* | At1g53170 | *AtERF79* |
| LOCOs06g08340.1 | *OsERF80* | Solyc06g082590.1.1 | | *SlERF80* | At5g44210 | *AtERF80* |
| LOCOs06g09390.1 | *OsERF81* | Solyc07g042230.1.1 | | *SlERF81* | At1g28360 | *AtERF81* |
| LOCOs06g09717.1 | *OsERF82* | Solyc07g049490.1.1 | | *SlERF82* | At1g50640 | *AtERF82* |
| LOCOs06g09760.1 | *OsERF83* | Solyc07g053740.1.1 | | *SlERF83* | At3g20310 | *AtERF83* |
| LOCOs06g09790.1 | *OsERF84* | Solyc07g054220.1.1 | | *SlERF84* | At1g80580 | *AtERF84* |
| LOCOs06g09810.1 | *OsERF85* | Solyc07g064890.1.1 | | *SlERF85* | At5g13910 | *AtERF85* |
| LOCOs06g10780.1 | *OsERF86* | Solyc08g007230.1.1 | | *SlERF86* | At5g18560 | *AtERF86* |
| LOCOs06g11860.1 | *OsERF87* | Solyc08g007820.1.1 | | *SlERF87* | At1g28160 | *AtERF87* |
| LOCOs06g11940.1 | *OsERF88* | Solyc08g007830.1.1 | | *SlERF88* | At1g12890 | *AtERF88* |
| LOCOs06g36000.1 | *OsERF89* | Solyc08g066660.1.1 | | *SlERF89* | At1g12980 | *AtERF89* |
| LOCOs06g40150.1 | *OsERF90* | Solyc08g078170.1.1 | | *SlERF90* | At1g24590 | *AtERF90* |
| LOCOs06g42990.1 | *OsERF91* | Solyc08g078180.1.1 | | *SlERF91* | At4g18450 | *AtERF91* |
| LOCOs06g44750.1 | *OsERF92* | Solyc08g078190.1.1 | | *SlERF92* | At3g23240 | *AtERF92* |
| LOCOs06g47590.1 | *OsERF93* | Solyc08g078410.1.1 | | *SlERF93* | At2g31230 | *AtERF93* |
| LOCOs07g03250.1 | *OsERF94* | Solyc08g078420.1.1 | | *SlERF94* | At1g06160 | *AtERF94* |
| LOCOs07g10410.1 | *OsERF95* | Solyc08g080290.2.1 | | *SlERF95* | At3g23220 | *AtERF95* |
| LOCOs07g12510.1 | *OsERF96* | Solyc08g081960.1.1 | | *SlERF96* | At5g43410 | *AtERF96* |
| LOCOs07g22730.1 | *OsERF97* | Solyc08g082210.2.1 | | *SlERF97* | At1g04370 | *AtERF97* |
| LOCOs07g22770.1 | *OsERF98* | Solyc09g009240.1.1 | | *SlERF98* | At3g23230 | *AtERF98* |
| LOCOs07g38750.1 | *OsERF99* | Solyc09g059510.2.1 | | *SlERF99* | At2g44840 | *AtERF99* |
| LOCOs07g42510.1 | *OsERF100* | Solyc09g066340.1.1 | | *SlERF100* | At4g17500 | *AtERF100* |
| LOCOs07g47330.1 | *OsERF101* | Solyc09g066350.1.1 | | *SlERF101* | At5g47220 | *AtERF101* |
| LOCOs07g47790.1 | *OsERF102* | Solyc09g066360.1.1 | | *SlERF102* | At5g47230 | *AtERF102* |
| LOCOs08g07700.1 | *OsERF103* | Solyc09g075420.2.1 | | *SlERF103* | At4g17490 | *AtERF103* |
| LOCOs08g27220.1 | *OsERF104* | Solyc09g089910.1.1 | | *SlERF104* | At5g61600 | *AtERF104* |
| LOCOs08g31580.1 | *OsERF105* | Solyc09g089920.1.1 | | *SlERF105* | At5g51190 | *AtERF105* |
| LOCOs08g34360.1 | *OsERF106* | Solyc09g089930.1.1 | | *SlERF106* | At5g07580 | *AtERF106* |
| LOCOs08g35240.1 | *OsERF107* | Solyc09g091950.1.1 | | *SlERF107* | At5g61590 | *AtERF107* |
| LOCOs08g36920.1 | *OsERF108* | Solyc10g006130.1.1 | | *SlERF108* | At1g43160 | *AtERF108* |
| LOCOs08g41030.1 | *OsERF109* | Solyc10g009110.1.1 | | *SlERF109* | At4g34410 | *AtERF109* |
| LOCOs08g42550.1 | *OsERF110* | Solyc10g050970.1.1 | | *SlERF110* | At5g50080 | *AtERF110* |
| LOCOs08g43200.1 | *OsERF111* | Solyc10g076370.1.1 | | *SlERF111* | At5g64750 | *AtERF111* |
| LOCOs08g43210.1 | *OsERF112* | Solyc10g076380.1.1 | | *SlERF112* | At2g33710 | *AtERF112* |
| LOCOs08g44960.1 | *OsERF113* | Solyc10g078610.1.1 | | *SlERF113* | At5g13330 | *AtERF113* |
| LOCOs08g45110.1 | *OsERF114* | Solyc10g080310.1.1 | | *SlERF114* | At5g61890 | *AtERF114* |
| LOCOs09g11460.1 | *OsERF115* | Solyc10g080650.1.1 | | *SlERF115* | At5g07310 | *AtERF115* |
| LOCOs09g11480.2 | *OsERF116* | Solyc10g083560.1.1 | | *SlERF116* | At1g25470.1 | *AtERF116* |
| LOCOs09g13940.1 | *OsERF117* | Solyc11g006050.1.1 | | *SlERF117* | At1g49120 | *AtERF117* |
| LOCOs09g20350.1 | *OsERF118* | Solyc11g011740.1.1 | | *SlERF118* | At1g68550.1 | *AtERF118* |
| LOCOs09g26420.1 | *OsERF119* | Solyc11g011750.1.1 | | *SlERF119* | At3g25890 | *AtERF119* |
| LOCOs09g28440.1 | *OsERF120* | Solyc11g012980.1.1 | | *SlERF120* | At2g20350 | *AtERF120* |
| LOCOs09g35010.1 | *OsERF121* | Solyc11g042560.1.1 | | *SlERF121* | At5g67010 | *AtERF121* |
| LOCOs09g35020.1 | *OsERF122* | Solyc11g042580.1.1 | | *SlERF122* | At5g67000 | *AtERF122* |
| LOCOs09g35030.1 | *OsERF123* | Solyc11g045680.1.1 | | *SlERF123* |  |  |
| LOCOs09g39810.1 | *OsERF124* | Solyc11g045690.1.1 | | *SlERF124* |  |  |
| LOCOs09g39850.1 | *OsERF125* | Solyc12g005960.1.1 | | *SlERF125* |  |  |
| LOCOs10g22600.1 | *OsERF126* | Solyc12g008350.1.1 | | *SlERF126* |  |  |
| LOCOs10g25170.1 | *OsERF127* | Solyc12g009240.1.1 | | *SlERF127* |  |  |
| LOCOs10g26590.1 | *OsERF128* | Solyc12g009490.1.1 | | *SlERF128* |  |  |
| LOCOs10g30840.1 | *OsERF129* | Solyc12g013660.1.1 | | *SlERF129* |  |  |
| LOCOs10g38000.1 | *OsERF130* | Solyc12g038440.1.1 | | *SlERF130* |  |  |
| LOCOs10g41130.1 | *OsERF131* | Solyc12g038450.1.1 | | *SlERF131* |  |  |
| LOCOs10g41330.1 | *OsERF132* | Solyc12g042210.1.1 | | *SlERF132* |  |  |
| LOCOs11g06770.2 | *OsERF133* | Solyc12g044390.1.1 | | *SlERF133* |  |  |
| LOCOs11g13840.1 | *OsERF134* | Solyc12g049560.1.1 | | *SlERF134* |  |  |
| LOCOs12g39330.1 | *OsERF135* | Solyc12g056430.1.1 | | *SlERF135* |  |  |
| LOCOs12g40960.1 | *OsERF136* | Solyc12g056590.1.1 | | *SlERF136* |  |  |
| LOCOs12g41030.1 | *OsERF137* | Solyc12g056980.1.1 | | *SlERF137* |  |  |
| LOCOs12g41060.1 | *OsERF138* |  | |  |  |  |

**Table S2.** List of primers used in real-time quantitative PCR (qPCR).

| **Gene name** | **Primer name** | **Sequence** |
| --- | --- | --- |
| *CaERF66* | CaERF66-F | AGAAGGTCGAAAGAGGAT |
|  | CaERF66-R | GCTGAGGAATTAGGGTTA |
| *CaERF82* | CaERF82-F | TAGCAACCTCTGCAGCTTCC |
|  | CaERF82-R | GCTGCTGCTACCTCCTTGAT |
| *CaERF97* | CaERF97-F | GTCGGTGTTCCTCCTCAACA |
|  | CaERF97-R | CATCTGTGAGGGGTGCTTGT |
| *CaERF101* | CaERF101-F | TCTGCTTCAAGACGTGCTGT |
|  | CaERF101-R | AATCATCAGATCCGCCGTCC |
| *CaERF107* | CaERF107-F | CACGACGAGGAGTCAGCAAT |
|  | CaERF107-R | TCGCGTCCAAATCATCCCAA |
| *CaERF28* | CaERF28-F | GCGAGGCTATGGCTAGGAAC |
|  | CaERF28-R | CAGAGGAGGAAGTCGATGGC |
| *CaERF53* | CaERF53-F | ATCATTCCTCGTCGTGACCG |
|  | CaERF53-R | TGCCCATTTACCCCATGGAC |
| *CaERF92* | CaERF92-F | AATGGATTGTCACCAGCAGCA |
|  | CaERF92-R | TACCCAGACAAGAGTTCGTCCA |
| *CaERF102* | CaERF102-F | CGCATTTGGCTTGGCACATA |
|  | CaERF102-R | GCTTTAGCCGCAGCATCTTG |
| *CaERF111* | CaERF111-F | GCAGCAGCAATGGACAAGTT |
|  | CaERF111-R | GTTGCCAGGTCTATTGCGGA |
| *CA00g52149* | CA00g52149-F | GGTCGCTTGGTTATGGTTAT |
|  | CA00g52149-R | ACAGTAGGGTCTCGGTTTGA |
| *CA12g20490* | CA12g20490-F | GAAGACCCTGACGGGCAAGAC |
|  | CA12g20490-R | TTAGCACCACCACGGAGACGA |

**Table S3.** *CaERF* genes identified and characterized in pepper.

| **Name** | **Chr** | **Position** | **Length** | **MW** | **pI** | **Instability** | **Corresponding gene ID** |
| --- | --- | --- | --- | --- | --- | --- | --- |
|  |  |  | **(aa)** | **(kDa)** |  | **index (II)** | **in CM334** |
| *CaERF1* | Chr01 | 2098453-2099364 | 303 | 34.15644 | 8.6 | 47.94 | CA.PGAv.1.6.scaffold532.114 |
| *CaERF2* | Chr01 | 2462644-2463669 | 341 | 37.7138 | 5.1 | 45.61 | CA.PGAv.1.6.scaffold532.90 |
| *CaERF3* | Chr01 | 7086801-7087989 | 239 | 26.1936 | 4.8963 | 78.66 | CA.PGAv.1.6.scaffold981.37 |
| *CaERF4* | Chr01 | 12816182-12816844 | 220 | 25.18545 | 5.65 | 48.77 | CA.PGAv.1.6.scaffold1178.18 |
| *CaERF5* | Chr01 | 12878972-12879661 | 229 | 25.49045 | 6.07 | 60.88 | CA.PGAv.1.6.scaffold1178.16 |
| *CaERF6* | Chr01 | 14099749-14100419 | 201 | 22.63275 | 4.53 | 62.75 | CA.PGAv.1.6.scaffold1856.9 |
| *CaERF7* | Chr01 | 57268218-57269135 | 305 | 33.7838 | 8.45 | 49.79 | CA.PGAv.1.6.scaffold170.49 |
| *CaERF8* | Chr01 | 63713054-63713683 | 209 | 23.754 | 9.3223 | 63.31 | CA.PGAv.1.6.scaffold362.3 |
| *CaERF9* | Chr01 | 75652864-75668895 | 268 | 30.58075 | 8.32 | 49.91 | CA.PGAv.1.6.scaffold379.18 |
| *CaERF10* | Chr01 | 75694500-75694880 | 126 | 14.53863 | 9.58 | 52.37 | CA.PGAv.1.6.scaffold379.17 |
| *CaERF11* | Chr01 | 75824707-75826500 | 146 | 17.0177 | 9.48 | 41.42 | CA.PGAv.1.6.scaffold379.14 |
| *CaERF12* | Chr01 | 75870555-75870965 | 136 | 16.11645 | 10 | 47.27 | CA.PGAv.1.6.scaffold379.13 |
| *CaERF13* | Chr01 | 75925253-75925759 | 168 | 18.75217 | 5.37 | 50.14 | CA.PGAv.1.6.scaffold379.12 |
| *CaERF14* | Chr01 | 76053789-76054169 | 126 | 14.65473 | 9.45 | 52 | CA.PGAv.1.6.scaffold379.9 |
| *CaERF15* | Chr01 | 76295749-76296315 | 188 | 21.24211 | 8.85 | 51.08 | CA.PGAv.1.6.scaffold379.8 |
| *CaERF16* | Chr01 | 76310086-76310490 | 134 | 15.61202 | 9.87 | 51.28 | CA.PGAv.1.6.scaffold379.7 |
| *CaERF17* | Chr01 | 76399800-76400306 | 168 | 18.9656 | 6.2 | 46.13 | CA.PGAv.1.6.scaffold379.6 |
| *CaERF18* | Chr01 | 76496647-76497051 | 134 | 15.67992 | 9.3 | 59.69 | CA.PGAv.1.6.scaffold379.4 |
| *CaERF19* | Chr01 | 76563918-76564127 | 69 | 7.85987 | 9.69 | 50.35 | CA.PGAv.1.6.scaffold379.3 |
| *CaERF20* | Chr01 | 81356637-81360856 | 257 | 29.87964 | 9.53 | 42.13 | CA.PGAv.1.6.scaffold64.8 |
| *CaERF21* | Chr01 | 81505722-81506299 | 166 | 19.02397 | 9.66 | 49.05 | CA.PGAv.1.6.scaffold64.6 |
| *CaERF22* | Chr01 | 126467683-126472417 | 266 | 29.3786 | 5.55 | 39.92 | CA.PGAv.1.6.scaffold14.17 |
| *CaERF23* | Chr01 | 179021541-179022194 | 217 | 24.79191 | 6.11 | 54.34 | CA.PGAv.1.6.scaffold386.28 |
| *CaERF24* | Chr01 | 234495547-234496614 | 355 | 39.97792 | 5.21 | 57.56 | CA.PGAv.1.6.scaffold223.23 |
| *CaERF25* | Chr01 | 252841508-252842098 | 196 | 22.14661 | 5.24 | 51.61 | CA.PGAv.1.6.scaffold1473.19 |
| *CaERF26* | Chr01 | 252860840-252861463 | 207 | 23.08146 | 7.96 | 59.4 | CA.PGAv.1.6.scaffold1473.20 |
| *CaERF27* | Chr01 | 252866151-252866687 | 178 | 20.57299 | 10.13 | 89.5 | CA.PGAv.1.6.scaffold1473.21 |
| *CaERF28* | Chr01 | 252872602-252873225 | 207 | 23.2829 | 7.83 | 76.83 | CA.PGAv.1.6.scaffold1473.22 |
| *CaERF29* | Chr01 | 252885626-252886303 | 225 | 24.89117 | 8.83 | 69.67 | CA.PGAv.1.6.scaffold1473.23 |
| *CaERF30* | Chr01 | 253411018-253411575 | 185 | 20.51212 | 9.65 | 42.9 | CA.PGAv.1.6.scaffold899.33 |
| *CaERF31* | Chr01 | 306179179-306180111 | 310 | 35.0621 | 5.84 | 52.3 | CA.PGAv.1.6.scaffold792.41 |
| *CaERF32* | Chr01 | 307110240-307110878 | 212 | 24.10521 | 8.77 | 52.4 | CA.PGAv.1.6.scaffold1394.6 |
| *CaERF33* | Chr01 | 307117802-307118536 | 244 | 27.48446 | 5.24 | 59.77 | CA.PGAv.1.6.scaffold1394.4 |
| *CaERF34* | Chr01 | 307121248-307121988 | 246 | 27.72994 | 6.14 | 49.51 | CA.PGAv.1.6.scaffold1394.3 |
| *CaERF35* | Chr02 | 12825962-12827274 | 242 | 27.52355 | 9.35 | 34.09 | CA.PGAv.1.6.scaffold408.6 |
| *CaERF36* | Chr02 | 16214134-16214604 | 156 | 17.62745 | 8.07 | 53.8 | CA.PGAv.1.6.scaffold291.8 |
| *CaERF37* | Chr02 | 129157277-129158026 | 249 | 28.75718 | 5.08 | 48.76 | CA.PGAv.1.6.scaffold79.70 |
| *CaERF38* | Chr02 | 129161667-129162209 | 180 | 20.34982 | 8.58 | 45.6 | CA.PGAv.1.6.scaffold79.71 |
| *CaERF39* | Chr02 | 129839017-129839727 | 236 | 26.70977 | 5.75 | 45.43 | CA.PGAv.1.6.scaffold660.1 |
| *CaERF40* | Chr02 | 129861376-129862131 | 251 | 27.96413 | 5.15 | 46.19 | CA.PGAv.1.6.scaffold660.3 |
| *CaERF41* | Chr02 | 138645779-138646279 | 166 | 18.42278 | 7.73 | 41.59 | CA.PGAv.1.6.scaffold370.34 |
| *CaERF42* | Chr02 | 138678372-138680943 | 512 | 56.78483 | 5.96 | 51.41 | CA.PGAv.1.6.scaffold370.36 |
| *CaERF43* | Chr02 | 141217850-141218491 | 157 | 17.16269 | 9.81 | 69.58 | CA.PGAv.1.6.scaffold529.31 |
| *CaERF44* | Chr02 | 141296624-141304606 | 639 | 70.58251 | 8.25 | 41.23 | CA.PGAv.1.6.scaffold529.35 |
| *CaERF45* | Chr02 | 141311171-141311671 | 166 | 18.4149 | 5.69 | 53.55 | CA.PGAv.1.6.scaffold529.36 |
| *CaERF46* | Chr02 | 141360857-141361468 | 203 | 22.85597 | 6.75 | 50.65 | CA.PGAv.1.6.scaffold529.37 |
| *CaERF47* | Chr02 | 141470407-141473062 | 303 | 33.78505 | 9.49 | 38.81 | CA.PGAv.1.6.scaffold529.39 |
| *CaERF48* | Chr02 | 155426778-155427498 | 209 | 23.2238 | 9.47 | 36.62 | CA.PGAv.1.6.scaffold358.83 |
| *CaERF49* | Chr03 | 5968581-5969351 | 256 | 28.65218 | 9.34 | 43.9 | CA.PGAv.1.6.scaffold1635.8 |
| *CaERF50* | Chr03 | 16623151-16623873 | 240 | 26.97694 | 5.35 | 62.82 | CA.PGAv.1.6.scaffold793.38 |
| *CaERF51* | Chr03 | 16657226-16657642 | 138 | 15.63267 | 8.79 | 65.31 | CA.PGAv.1.6.scaffold793.37 |
| *CaERF52* | Chr03 | 16682157-16682576 | 139 | 15.25269 | 6.31 | 60.18 | CA.PGAv.1.6.scaffold793.36 |
| *CaERF53* | Chr03 | 28667513-28668567 | 264 | 30.05665 | 5.53 | 48.87 | CA.PGAv.1.6.scaffold862.63 |
| *CaERF54* | Chr03 | 53263694-53264626 | 310 | 33.9672 | 5.73 | 55.13 | CA.PGAv.1.6.scaffold156.10 |
| *CaERF55* | Chr03 | 107266401-107267219 | 272 | 30.59146 | 7.63 | 51.08 | CA.PGAv.1.6.scaffold247.16 |
| *CaERF56* | Chr03 | 129506865-129507455 | 196 | 22.48337 | 5.17 | 53.05 | CA.PGAv.1.6.scaffold1570.1 |
| *CaERF57* | Chr03 | 138205619-138206044 | 141 | 16.03754 | 5.87 | 48 | CA.PGAv.1.6.scaffold977.10 |
| *CaERF58* | Chr03 | 150329943-150330137 | 64 | 7.52248 | 9.8 | 61.64 | CA.PGAv.1.6.scaffold1691.1 |
| *CaERF59* | Chr03 | 222356416-222356886 | 156 | 17.53955 | 10.16 | 56.88 | CA.PGAv.1.6.scaffold548.13 |
| *CaERF60* | Chr03 | 222583149-222583475 | 108 | 12.13281 | 10.64 | 52.77 | CA.PGAv.1.6.scaffold548.15 |
| *CaERF61* | Chr03 | 243311495-243312682 | 395 | 44.55231 | 4.51 | 59.54 | CA.PGAv.1.6.scaffold108.20 |
| *CaERF62* | Chr03 | 268446852-268447505 | 217 | 24.65569 | 9.31 | 57.67 | CA.PGAv.1.6.scaffold982.3 |
| *CaERF63* | Chr03 | 273629223-273630071 | 202 | 22.45143 | 8.9 | 64.91 | CA.PGAv.1.6.scaffold407.93 |
| *CaERF64* | Chr03 | 274496627-274497553 | 163 | 18.4415 | 5.42 | 59.5 | CA.PGAv.1.6.scaffold407.46 |
| *CaERF65* | Chr03 | 274616734-274617549 | 271 | 30.3248 | 9.35 | 41.89 | CA.PGAv.1.6.scaffold407.38 |
| *CaERF66* | Chr03 | 276523089-276524660 | 284 | 31.93855 | 6.22 | 58.91 | CA.PGAv.1.6.scaffold843.11 |
| *CaERF67* | Chr03 | 278641807-278643057 | 416 | 46.56051 | 4.77 | 45.18 | CA.PGAv.1.6.scaffold438.135 |
| *CaERF68* | Chr03 | 279152797-279158666 | 469 | 52.60026 | 9.02 | 45.76 | CA.PGAv.1.6.scaffold438.111 |
| *CaERF69* | Chr03 | 280595318-280596118 | 266 | 29.05521 | 6.15 | 64.53 | CA.PGAv.1.6.scaffold438.8 |
| *CaERF70* | Chr03 | 281843842-281846167 | 377 | 41.57769 | 4.9 | 36.83 | CA.PGAv.1.6.scaffold790.71 |
| *CaERF71* | Chr04 | 544631-545026 | 131 | 14.99541 | 5.9 | 42.02 | CA.PGAv.1.6.scaffold638.61 |
| *CaERF72* | Chr04 | 565105-565644 | 179 | 20.36296 | 8.4 | 42.68 | CA.PGAv.1.6.scaffold638.60 |
| *CaERF73* | Chr04 | 4505281-4505607 | 108 | 11.65995 | 6.74 | 86.62 | CA.PGAv.1.6.scaffold189.63 |
| *CaERF74* | Chr04 | 4571879-4572202 | 107 | 11.53703 | 9.34 | 51.79 | CA.PGAv.1.6.scaffold189.62 |
| *CaERF75* | Chr04 | 4835779-4836360 | 193 | 21.22209 | 7.1 | 28.69 | CA.PGAv.1.6.scaffold189.55 |
| *CaERF76* | Chr04 | 4926584-4927213 | 209 | 22.95999 | 9.12 | 51.22 | CA.PGAv.1.6.scaffold189.53 |
| *CaERF77* | Chr04 | 4932666-4933151 | 135 | 14.92197 | 9.23 | 56.61 | CA.PGAv.1.6.scaffold189.52 |
| *CaERF78* | Chr04 | 4938791-4939063 | 90 | 10.29501 | 10.28 | 65.18 | CA.PGAv.1.6.scaffold189.51 |
| *CaERF79* | Chr04 | 28683090-28683548 | 152 | 16.22239 | 5.4 | 44.26 | CA.PGAv.1.6.scaffold568.1 |
| *CaERF80* | Chr04 | 46889950-46890693 | 247 | 27.03696 | 5.33 | 62.88 | CA.PGAv.1.6.scaffold127.3 |
| *CaERF81* | Chr04 | 90394046-90394564 | 172 | 19.14432 | 5.09 | 45.12 | CA.PGAv.1.6.scaffold101.2 |
| *CaERF82* | Chr04 | 198786713-198788417 | 450 | 49.10067 | 5.84 | 73.89 | CA.PGAv.1.6.scaffold264.14 |
| *CaERF83* | Chr04 | 206278304-206278777 | 157 | 17.64277 | 5.69 | 84.25 | CA.PGAv.1.6.scaffold1010.16 |
| *CaERF84* | Chr04 | 211971421-211972143 | 77 | 35.3405 | 9.0106 | 54.82 | CA.PGAv.1.6.scaffold517.1 |
| *CaERF85* | Chr04 | 235345211-235346224 | 337 | 37.21715 | 4.89 | 38.58 | CA.PGAv.1.6.scaffold851.27 |
| *CaERF86* | Chr05 | 4652604-4653464 | 286 | 32.68335 | 4.73 | 53.17 | CA.PGAv.1.6.scaffold525.7 |
| *CaERF87* | Chr05 | 6703606-6705611 | 394 | 43.88303 | 6.01 | 52.66 | CA.PGAv.1.6.scaffold787.27 |
| *CaERF88* | Chr05 | 17488033-17489128 | 297 | 33.16413 | 5.48 | 52.59 | CA.PGAv.1.6.scaffold624.7 |
| *CaERF89* | Chr05 | 17495854-17497028 | 309 | 34.44737 | 5.37 | 46.97 | CA.PGAv.1.6.scaffold624.6 |
| *CaERF90* | Chr05 | 180027897-180028466 | 189 | 21.62155 | 10.04 | 67.85 | CA.PGAv.1.6.scaffold365.10 |
| *CaERF91* | Chr05 | 185095934-185096644 | 236 | 27.12314 | 5.63 | 63.53 | CA.PGAv.1.6.scaffold112.1 |
| *CaERF92* | Chr05 | 185232361-185232885 | 174 | 19.62091 | 7.73 | 57.21 | CA.PGAv.1.6.scaffold112.2 |
| *CaERF93* | Chr05 | 188884560-188885123 | 187 | 21.63844 | 9.71 | 56.37 | CA.PGAv.1.6.scaffold112.26 |
| *CaERF94* | Chr05 | 207993911-207994741 | 276 | 31.38927 | 6.78 | 49.32 | CA.PGAv.1.6.scaffold296.14 |
| *CaERF95* | Chr05 | 208049394-208050215 | 273 | 30.63394 | 8.65 | 64.7 | CA.PGAv.1.6.scaffold296.13 |
| *CaERF96* | Chr05 | 210853815-210854234 | 139 | 15.5476 | 9.88 | 56.08 | CA.PGAv.1.6.scaffold1895.1 |
| *CaERF97* | Chr05 | 222845741-222846871 | 376 | 41.68538 | 5.03 | 44.65 | CA.PGAv.1.6.scaffold203.8 |
| *CaERF98* | Chr06 | 89485409-89486035 | 208 | 22.3783 | 4.2419 | 55.48 | CA.PGAv.1.6.scaffold28.15 |
| *CaERF99* | Chr06 | 117390829-117391947 | 372 | 42.10582 | 4.93 | 50.19 | CA.PGAv.1.6.scaffold589.15 |
| *CaERF100* | Chr06 | 203718100-203719129 | 194 | 22.28955 | 8.4 | 53.98 | CA.PGAv.1.6.scaffold199.69 |
| *CaERF101* | Chr06 | 210593146-210595272 | 375 | 41.58584 | 4.82 | 41.47 | CA.PGAv.1.6.scaffold1743.2 |
| *CaERF102* | Chr06 | 216737633-216738208 | 191 | 21.50423 | 5.33 | 39.92 | CA.PGAv.1.6.scaffold1152.16 |
| *CaERF103* | Chr06 | 219891138-219891757 | 176 | 20.09039 | 7.02 | 57.78 | CA.PGAv.1.6.scaffold1490.12 |
| *CaERF104* | Chr06 | 239397220-239397972 | 250 | 28.37468 | 5.21 | 43.47 | CA.PGAv.1.6.scaffold874.70 |
| *CaERF105* | Chr07 | 18321224-18322327 | 367 | 40.40332 | 4.71 | 52.99 | CA.PGAv.1.6.scaffold1258.1 |
| *CaERF106* | Chr07 | 205045921-205046547 | 208 | 23.14494 | 6.22 | 54.03 | CA.PGAv.1.6.scaffold290.20 |
| *CaERF107* | Chr07 | 222934983-222936251 | 422 | 47.42646 | 5.26 | 77.54 | CA.PGAv.1.6.scaffold248.38 |
| *CaERF108* | Chr07 | 225971329-225971763 | 144 | 15.59358 | 9.79 | 51.88 | CA.PGAv.1.6.scaffold209.24 |
| *CaERF109* | Chr07 | 246264453-246278166 | 242 | 27.04201 | 5.68 | 53.87 | CA.PGAv.1.6.scaffold337.65 |
| *CaERF110* | Chr08 | 3120068-3125322 | 309 | 35.23566 | 8.24 | 39.09 | CA.PGAv.1.6.scaffold1834.3 |
| *CaERF111* | Chr08 | 92186955-92187626 | 223 | 24.44138 | 6.16 | 49.26 | CA.PGAv.1.6.scaffold335.16 |
| *CaERF112* | Chr08 | 135206770-135207651 | 293 | 33.8226 | 6.39 | 43.9 | CA.PGAv.1.6.scaffold986.47 |
| *CaERF113* | Chr09 | 35128371-35130407 | 140 | 15.9479 | 8.83 | 30.68 | CA.PGAv.1.6.scaffold554.3 |
| *CaERF114* | Chr09 | 67050993-67060604 | 515 | 59.2693 | 4.87 | 51.75 | CA.PGAv.1.6.scaffold967.3 |
| *CaERF115* | Chr09 | 243108826-243109962 | 378 | 40.33092 | 6.02 | 43.24 | CA.PGAv.1.6.scaffold1698.1 |
| *CaERF116* | Chr09 | 266938355-266938918 | 187 | 20.8778 | 6.7492 | 69.05 | CA.PGAv.1.6.scaffold1661.27 |
| *CaERF117* | Chr10 | 1474410-1474985 | 191 | 21.68786 | 11.1 | 42.6 | CA.PGAv.1.6.scaffold492.22 |
| *CaERF118* | Chr10 | 85970961-85977946 | 280 | 31.59893 | 9.62 | 39.37 | CA.PGAv.1.6.scaffold332.7 |
| *CaERF119* | Chr10 | 151547195-151548691 | 452 | 51.90323 | 4.6 | 66.46 | CA.PGAv.1.6.scaffold1225.2 |
| *CaERF120* | Chr10 | 219668126-219668923 | 193 | 20.2304 | 5.68 | 48.4 | CA.PGAv.1.6.scaffold1138.21 |
| *CaERF121* | Chr10 | 229384953-229386143 | 396 | 44.32061 | 4.42 | 48.39 | CA.PGAv.1.6.scaffold128.60 |
| *CaERF122* | Chr10 | 230056321-230057250 | 309 | 34.71054 | 6.18 | 44.31 | CA.PGAv.1.6.scaffold128.18 |
| *CaERF123* | Chr11 | 4507980-4509041 | 353 | 38.92529 | 4.72 | 37.58 | CA.PGAv.1.6.scaffold656.14 |
| *CaERF124* | Chr11 | 5260485-5260898 | 137 | 14.9804 | 6.58 | 43.58 | CA.PGAv.1.6.scaffold1982.7 |
| *CaERF125* | Chr11 | 120297792-120299003 | 403 | 44.47055 | 8.44 | 57.5 | CA.PGAv.1.6.scaffold493.3 |
| *CaERF126* | Chr11 | 248448452-248449350 | 271 | 30.41818 | 4.78 | 51.26 | CA.PGAv.1.6.scaffold1187.1 |
| *CaERF127* | Chr12 | 5245617-5246123 | 168 | 19.29485 | 5.99 | 54.25 | CA.PGAv.1.6.scaffold222.137 |
| *CaERF128* | Chr12 | 5267125-5267475 | 116 | 13.47577 | 4.64 | 60.42 | CA.PGAv.1.6.scaffold222.138 |
| *CaERF129* | Chr12 | 5279891-5297660 | 282 | 32.10678 | 9.22 | 51.24 | CA.PGAv.1.6.scaffold222.139 |
| *CaERF130* | Chr12 | 5368921-5369427 | 168 | 19.34184 | 5.52 | 52.68 | CA.PGAv.1.6.scaffold222.142 |
| *CaERF131* | Chr12 | 5546297-5546689 | 130 | 14.91769 | 5.4 | 52.95 | CA.PGAv.1.6.scaffold222.145 |
| *CaERF132* | Chr12 | 35721784-35722371 | 195 | 22.26294 | 4.95 | 55.22 | CA.PGAv.1.6.scaffold582.14 |
| *CaERF133* | Chr12 | 49800059-49801868 | 332 | 37.9334 | 6.68 | 58.49 | CA.PGAv.1.6.scaffold452.3 |
| *CaERF134* | Chr12 | 83965441-83966019 | 192 | 21.21071 | 4.86 | 51.41 | CA.PGAv.1.6.scaffold4.16 |
| *CaERF135* | Chr12 | 178488628-178490331 | 269 | 30.53953 | 6.9 | 56.16 | CA.PGAv.1.6.scaffold1452.2 |
| *CaERF136* | Chr12 | 201811727-201812395 | 222 | 24.94767 | 4.89 | 49.97 | CA.PGAv.1.6.scaffold26.31 |
| *CaERF137* | Chr12 | 210361768-210390829 | 449 | 49.76505 | 4.92 | 44.28 | CA.PGAv.1.6.scaffold390.43 |
| *CaERF138* | Chr00 | 184864-185769 | 301 | 33.05345 | 7.63 | 54.45 | CA.PGAv.1.6.scaffold1041.13 |
| *CaERF139* | Chr00 | 222592-222885 | 97 | 11.28695 | 9.55 | 52.52 | CA.PGAv.1.6.scaffold1211.2 |
| *CaERF140* | Chr00 | 416324-423090 | 672 | 74.91157 | 6.84 | 39.69 | CA.PGAv.1.6.scaffold960.44 |
| *CaERF141* | Chr00 | 496822-498697 | 287 | 31.94975 | 6.46 | 52.2 | CA.PGAv.1.6.scaffold1089.24 |
| *CaERF142* | Chr00 | 675757-676206 | 149 | 16.92206 | 9.07 | 54.54 | CA.PGAv.1.6.scaffold1030.30 |

Five *CaERFs* (*CaERF138-CaERF14*2) could not be located on any of the pepper chromosomes. They were thus arranged them on a putative-chromosome, which were designated as Chr00.

**Table S4.** Biological functions of characterized ERF proteins that potentially exist in *Arabidopsis* and tomato.

| **Name** | **Subgroup** | **General function** | **Specific function** | **References** |
| --- | --- | --- | --- | --- |
| AtERF39 | IV | Cell walls development | Primary cell walls and secondary cell walls formation | [1, 2, 3] |
| AtERF40 |  |  |  |  |
| AtERF43 |  |  |  |  |
| AtERF44 |  |  |  |  |
| AtERF24 | III | Defense responses | A negative regulator of pattern-triggered immunity | [4] |
| AtERF78 | IXa | Development | Regulation of leaf senescence Regulation of anthocyanin biosynthesis | [5]  [6] |
| AtERF79 | IXa |  |  |  |
| SlERF40 | Xa |  | Regulation of flower pedicel abscission development | [7] |
| SlERF8 | VIII | Development | Regulation of steroidal glycoalkaloids biosynthesis | [8]  [9]  [10] |
| SlERF9 |  |  |  |  |
| SlERF10 |  |  |  |  |
| SlERF11 |  |  |  |  |
| SlERF12 |  |  |  |  |
| SlERF62 |  |  |  |  |
| SlERF91 | VIII | Development | Regulation of hypersensitive cell death and disease defense | [11] |
| SlERF66 | VIII | Response to stress tolerance  Development | Response to cold and salt stress  Regulation of carotenoid | [12, 13] |
| SlERF6 | VII | Development | Regulation of carotenoid biosynthesis and fruit ripening | [14] |
| SlERF56 | I |  | A component of the central ABA response | [15] |
| SlERF54 | V | Response to stress tolerance | Response to drought and salt stress Response to pathogen *Pst DC3000* | [16] |

**Table S5.** Putative CaERF homologs (version 2.0) of pepper ERF proteins with known biological functions.

| **Short name** | **Accession number** | **Putative CaERF (version 2.0)** | **Subgroup** | **Specific function** | **References** |
| --- | --- | --- | --- | --- | --- |
| Erf | KF060657 | CaERF53 | VII | Regulation of the pungency phenotype | [17] |
| Jerf | KF169944 | CaERF101 | VII | Regulation of the pungency phenotype | [17] |
| CaPF1 | AY246274 | CaERF101 | VII | Association with polyamine biosynthesis Response to freeze stress | [18, 19] |
| JERF1 | AY044235 | CaERF101 | VII | Regulation of ABA biosynthesis Response to freezing stress | [20] |
| CaPTI1 | KJ690096 | CaERF38 | VIII | Regulation of *P. capsici* defence | [21] |
| CaAIEF1 | KY652734 | CaERF38 | VIII | Positive regulation of drought stress and ABA signalling | [22] |

**Table S6.** Multilevel consensus sequence identiﬁed in 144 *CaERF* genes.

| **Motif** | **The sequence of conserved domains** | **E-value** | **Sites** | **Width** | **Logo** |
| --- | --- | --- | --- | --- | --- |
| 1 | GKWVAEIRDPRKKTRVWLGTFDTAEEAARAYD | 3.2e-2254 | 100 | 32 | 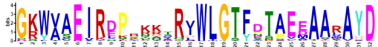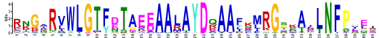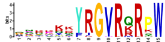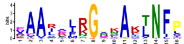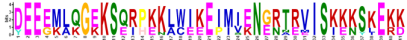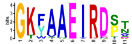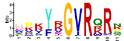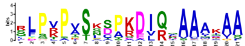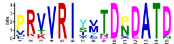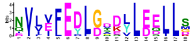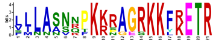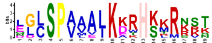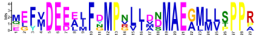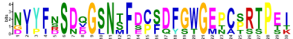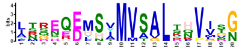 |
| 2 | RNGARVWLGTFDTAEEAALAYDQAAFKMRGSKAILNFPHEI | 4.2e-977 | 43 | 41 |  |
| 3 | QNKKKKYRGVRQRPW | 1.5e-732 | 100 | 15 |  |
| 4 | EAAREJRGPKAKTNFP | 1.3e-523 | 86 | 16 |  |
| 5 | DEEEMLQGEKSQRPKKLWIKEIMIENGRTRVISKKKSKEKK | 3.60E-203 | 10 | 41 |  |
| 6 | GKFAAEIRDPT | 2.90E-184 | 33 | 11 |  |
| 7 | HPKYRGVRQRN | 8.70E-123 | 37 | 11 |  |
| 8 | RLPVPVSKSPKDIQQAAAKAA | 2.50E-70 | 17 | 21 |  |
| 9 | PRVVRIYVTDPDATD | 2.60E-70 | 12 | 15 |  |
| 10 | NVLVFEDLGDDLLEELLS | 7.90E-68 | 13 | 18 |  |
| 11 | LLLASNNPKKRAGRKKFKETR | 6.20E-45 | 8 | 21 |  |
| 12 | EGLSPAAALKKRHKKRKST | 4.80E-33 | 9 | 19 |  |
| 13 | QEFVDEEEJFDMPNLJDBMAEGMLLSPPR | 3.10E-32 | 5 | 29 |  |
| 14 | NVYFNSDZGSNSFDCSDFGWGEPCPRTPEI | 2.00E-33 | 5 | 30 |  |
| 15 | LTREQEMSVMVSALTHVISG | 6.50E-32 | 10 | 20 |  |

**References**

1. Saelim L, Akiyoshi N, Tan TT, Ihara A, Yamaguchi M, Hirano K, Matsuoka M, Demura T, Ohtani M: *Arabidopsis* Group IIId ERF proteins positively regulate primary cell wall-type *CESA* genes. J Plant Res. 2019;132(1):117-129.

2. Hirano K, Aya K, Morinaka Y, Nagamatsu S, Sato Y, Antonio BA, Namiki N, Nagamura Y, Matsuoka M: Survey of genes involved in rice secondary cell wall formation through a co-expression network. Plant Cell Physiol. 2013;54(11):1803-1821.

3. Lasserre E, Jobet E, Llauro C, Delseny M: *AtERF38* (*At2g35700*), an AP2/ERF family transcription factor gene from *Arabidopsis thaliana*, is expressed in specific cell types of roots, stems and seeds that undergo suberization. Plant Physiol Bioch. 2008;46(12):1051-1061.

4. Huang PY, Zhang J, Jiang B, Chan C, Yu JH, Lu YP, Chung K, Zimmerli L: NINJA-associated ERF19 negatively regulates *Arabidopsis* pattern-triggered immunity. J Exp Bot 2019;70(3):1033-1047.

5. Koyama T, Sato F: The function of *ETHYLENE RESPONSE FACTOR* genes in the light-induced anthocyanin production of *Arabidopsis thaliana* leaves. Plant Biotechnol. 2018;35(1):87-91.

6. Koyama T, Nii H, Mitsuda N, Ohta M, Kitajima S, Ohme-Takagi M: A regulatory cascade involving class II ETHYLENE RESPONSE FACTOR transcriptional repressors operates in the progression of leaf senescence. Plant Physiol. 2013;162(2):991-1005.

7. Nakano T, Fujisawa M, Shima Y, Ito Y: The AP2/ERF transcription factor SlERF52 functions in flower pedicel abscission in tomato. J Exp Bot. 2014; 65(12): 3111–3119.

8. Thagun C, Imanishi S, Kudo T, Nakabayashi R, Ohyama K, Mori T, Kawamoto K, Nakamura Y, Katayama M, Nonaka S et al: Jasmonate-responsive ERF transcription factors regulate steroidal glycoalkaloid biosynthesis in tomato. Plant Cell Physiol. 2016;57(5):961-975.

9. Abdelkareem A, Thagun C, Nakayasu M, Mizutani M, Hashimoto T, Shoji T: Jasmonate-induced biosynthesis of steroidal glycoalkaloids depends on COI1 proteins in tomato. Biochem Bioph Res Co. 2017;489(2):206-210.

10. Nakayasu M, Shioya N, Shikata M, Shikata M, Thagun C, Thagun C, Abdelkareem A, Abdelkareem A, Okabe Y, Ariizumi T et al: JRE4 is a master transcriptional regulator of defense‐related steroidal glycoalkaloids in tomato. Plant J. 2018;94(6):975-990.

11. Liu AC, Cheng CP: Pathogen‐induced ERF68 regulates hypersensitive cell death in tomato. Mol Plant Pathol. 2017;18(8):1062-1074.

12. Klay I, Pirrello J, Riahi L, Bernadac A, Cherif A, Bouzayen M, Bouzid S: Ethylene response factor Sl-ERF.B.3 is responsive to abiotic stresses and mediates salt and cold stress response regulation in tomato. The Scientific World J. 2014; 2014:167681

13. Liu M, Diretto G, Pirrello J, Roustan J, Li Z, Giuliano G, Regad F, Bouzayen M: The chimeric repressor version of an *Ethylene Response Factor (ERF)* family member, *Sl-ERF.B3*, shows contrasting effects on tomato fruit ripening. New Phytol. 2014;203(1):206-218.

14. Lee JM, Joung JG, McQuinn R, Chung MY, Fei Z, Tieman D, Klee H, Giovannoni J: Combined transcriptome, genetic diversity and metabolite profiling in tomato fruit reveals that the ethylene response factor *SlERF6* plays an important role in ripening and carotenoid accumulation. Plant J 2012;70(2):191-204.

15. Upadhyay RK, Gupta A, Soni D, Garg R, Pathre UV, Nath P, Sane AP: Ectopic expression of a tomato DREB gene affects several ABA processes and influences plant growth and root architecture in an age-dependent manner. J Plant Physiol. 2017;48(2):97-107.

16. Peng R, Li Z, Gao J, Yao Q, Tian Y, Fu X, Wang B, Han H, Wang L, Xu J: A tomato ERF transcription factor, SlERF84, confers enhanced tolerance to drought and salt stress but negatively regulates immunity against *Pseudomonas syringae pv. tomato* DC3000. Plant Physiol Bioch. 2018;132:683-695.

17. Keyhaninejad N, Curry J, Romero J, O’Connell MA: Fruit specific variability in capsaicinoid accumulation and transcription of structural and regulatory genes in *Capsicum* fruit. Plant Sci. 2014;215-216:59-68.

18. Tang W, Newton RJ, Li C, Charles TM: Enhanced stress tolerance in transgenic pine expressing the pepper *CaPF1* gene is associated with the polyamine biosynthesis. Plant Cell Rep. 2007;26(1):115-124.

19. Yi SY, Kim JH, Joung YH, Lee S, Kim WT, Yu SH, Choi D: The pepper transcription factor CaPF1 confers pathogen and freezing tolerance in *Arabidopsis*. Plant Physiol. 2004;136(1):2862-2874.

20. Wu L, Chen X, Ren H, Zhang Z, Zhang H, Wang J, Wang XC, Huang R: ERF protein JERF1 that transcriptionally modulates the expression of abscisic acid biosynthesis-related gene enhances the tolerance under salinity and cold in tobacco. Planta. 2007;226(4):815-825.

21. Huang R, Li W, Guan XW, Xie B, Zhang SM: Molecular cloning and characterization of genes related to the ethylene signal transduction pathway in pomegranate (*Punica granatum* L.) under different temperature treatments. J Biosci. 2019;44(6):137.

22. Hong E, Lim CW, Han SW, Lee SC: Functional analysis of the pepper ethylene-responsive transcription factor, CaAIEF1, in enhanced ABA sensitivity and drought tolerance. Front Plant Sci. 2017;8:1407.
